# *In Vivo* Changes in Lamina Cribrosa Microarchitecture and Optic Nerve Head Structure in Early Experimental Glaucoma

**DOI:** 10.1371/journal.pone.0134223

**Published:** 2015-07-31

**Authors:** Kevin M. Ivers, Nripun Sredar, Nimesh B. Patel, Lakshmi Rajagopalan, Hope M. Queener, Michael D. Twa, Ronald S. Harwerth, Jason Porter

**Affiliations:** 1 College of Optometry, University of Houston, Houston, Texas, United States of America; 2 Department of Computer Science, University of Houston, Houston, Texas, United States of America; 3 School of Optometry, University of Alabama, Birmingham, Birmingham, Alabama, United States of America; University of Melbourne, AUSTRALIA

## Abstract

The lamina cribrosa likely plays an important role in retinal ganglion cell axon injury in glaucoma. We sought to (1) better understand optic nerve head (ONH) structure and anterior lamina cribrosa surface (ALCS) microarchitecture between fellow eyes of living, normal non-human primates and (2) characterize the time-course of *in vivo* structural changes in the ONH, ALCS microarchitecture, and retinal nerve fiber layer thickness (RNFLT) in non-human primate eyes with early experimental glaucoma (EG). Spectral domain optical coherence tomography (SDOCT) images of the ONH were acquired cross-sectionally in six bilaterally normal rhesus monkeys, and before and approximately every two weeks after inducing unilateral EG in seven rhesus monkeys. ONH parameters and RNFLT were quantified from segmented SDOCT images. Mean ALCS pore area, elongation and nearest neighbor distance (NND) were quantified globally, in sectors and regionally from adaptive optics scanning laser ophthalmoscope images. In bilaterally normal monkeys, ONH parameters were similar between fellow eyes with few inter-eye differences in ALCS pore parameters. In EG monkeys, an increase in mean ALCS Depth (ALCSD) was the first structural change measured in 6 of 7 EG eyes. A decrease in mean minimum rim width (MRW) simultaneously accompanied this early change in 4 of 6 EG eyes and was the first structural change in the 7^th^ EG eye. Mean ALCS pore parameters were among the first or second changes measured in 4 EG eyes. Mean ALCS pore area and NND increased in superotemporal and temporal sectors and in central and peripheral regions at the first time-point of change in ALCS pore geometry. RNFLT and/or mean ALCS radius of curvature were typically the last parameters to initially change. Survival analyses found mean ALCSD was the only parameter to significantly show an initial change prior to the first measured loss in RNFLT across EG eyes.

## Introduction

Glaucoma is a complex group of eye diseases that results in the death of retinal ganglion cells (RGCs) and degeneration of their axons, culminating in irreversible vision loss. The mechanisms responsible for the onset and progression of glaucoma are not well understood. Axonal damage is believed to initially occur in the optic nerve head (ONH) at the level of the lamina cribrosa (LC), a sieve-like structure comprised primarily of a meshwork of collagen beams that provides structural and functional support to RGC axons that pass through the ONH to the brain. Previous work has shown that increased stress from elevated intraocular pressure (IOP) can strain or deform the LC [1–3], resulting in biomechanical alterations to the load-bearing LC beams [4,5] and changes in LC beam and pore geometry [6]. Stretching and deformation of these beams (and associated pores) could shear or damage encompassed axons and capillaries, thereby hampering axonal transport [7–10], blood flow and the diffusion of nutrients [11], and the neurotrophic support provided to axons due to alterations in overlying astrocytes [12].

The objective clinical measure that has become the mainstay in glaucoma diagnosis and management is retinal nerve fiber layer thickness assessed in the circumpapillary region. However, recent work has shown that 10–15% of RGC axons are already lost when a first change in retinal nerve fiber layer thickness (RNFLT) is reliably detected [13]. Therefore, it is important to better understand structural alterations in early stages of glaucoma for earlier detection and diagnosis. Several *ex vivo* studies have shown a posterior displacement of the anterior lamina cribrosa surface (ALCS) in human glaucoma patients [14–16] and in non-human primates with early experimental glaucoma [4,17,18]. These histological observations have been confirmed *in vivo* in human glaucoma patients who have shown significantly larger mean anterior lamina cribrosa surface depths (ALCSDs) compared to normal eyes [19,20]. Increases in mean ALCSD have also been measured *in vivo* prior to a reduction in RNFLT in non-human primates with early experimental glaucoma [21]. Furthermore, significant decreases in mean minimum rim width (MRW), an ONH parameter that may potentially provide earlier detection of RNFL loss, and significant increases in mean ALCSD have been shown to precede a significant loss in RNFLT in glaucoma [22–25].

In addition to examining global ONH structure and LC shape, it is important to understand global and local changes in LC microarchitecture with disease [26–30]. Early histological studies in normal human eyes showed decreased connective tissue density and larger pore areas in the superior and inferior sectors of the LC and suggested this LC geometry could potentially be responsible for the regional susceptibility of RGC axons to early damage in glaucoma [31,32]. More recent histological work by Roberts et al. in normal non-human primate eyes found LC connective tissue to be most dense in the central and superior regions of the ONH [33]. However, despite an increase in the number of beams that were measured throughout the thickness of the LC in early experimental glaucoma eyes, the connective tissue volume fraction (the relative amount of beams within the LC volume) remained relatively unchanged [33]. In light of this work, *in vivo* studies are necessary to validate *ex vivo* results and examine longitudinal changes in LC microarchitecture during glaucoma.

High-resolution, *in vivo* measurements of the LC microarchitecture have been carried out in normal and glaucomatous eyes using a variety of imaging techniques (e.g., adaptive optics scanning laser ophthalmoscopy and optical coherence tomography) [6,34–38]. Larger ALCS pore areas have been measured at a single time-point in human glaucoma patients [35] and non-human primate eyes with experimental glaucoma [6,37] (compared to normal human subjects and fellow control eyes, respectively) via adaptive optics scanning laser ophthalmoscope (AOSLO) imaging. Three-dimensional images of the LC acquired using swept source optical coherence tomography (SSOCT) imaging have shown significantly increased beam thickness and significantly decreased pore diameter with increasingly worse visual field performance (i.e., worse mean deviation) in human glaucoma patients at single time-points [38]. These and other cross-sectional studies support the need to longitudinally examine the structural changes that occur in LC microarchitecture on global and local scales in concert with the structural changes that occur in the ONH and RNFL during the progression of glaucoma.

The main purpose of this study was to determine whether *in vivo* changes in LC pore and ONH structure precede changes in retinal nerve fiber layer thickness (RNFLT) in non-human primates with unilateral experimental glaucoma. We cross-sectionally examined ALCS pore microarchitecture and ONH structure in living, bilaterally normal non-human primate eyes and longitudinally examined ALCS pore microarchitecture, ONH structure, and RNFLT in non-human primates with unilateral experimental glaucoma. Statistical tests were performed to determine the initial onset of change in ALCS pore parameters, ONH parameters, and RNFLT. This paper provides a better understanding of the physiologic inter-eye differences in ALCS pore microarchitecture and ONH structure in normal eyes and of the time-course of structural changes in the ONH, ALCS pore microarchitecture, and RNFL in early experimental glaucoma.

## Methods

This study was carried out in accordance with the recommendations in the Guide for the Care and Use of Laboratory Animals of the National Research Council of the National Academy (2011 Guide) and was approved by the National Eye Institute (National Institutes of Health). The protocol and all animal care experimental procedures were approved by the University of Houston’s Institutional Animal Care and Use Committee (IACUC; Protocol Number: 12–010) and adhered to the ARVO Statement for the Use of Animals in Ophthalmic and Vision Research. All efforts were made to minimize potential suffering. Animals were housed in a dedicated non-human primate vivarium at the University of Houston (an accredited Association for Assessment and Accreditation of Laboratory Animal Care International [AAALAC] institution; USDA license 74-R-0020; OLAW assurance A3136-01) and were paired unless they were also subjects in ongoing behavioral experiments approved by the IACUC. Cage dimensions complied with the 2011 Guide’s recommendation for minimum space for Group 4 non-human primates (floor area of at least 6 sq ft and height of at least 32 in). Animals were fed twice daily with a hi-fiber primate diet (Harlan, 7195 Tekland Hi-Fiber Primate Diet). The IACUC approved environmental enrichment plan included five categories (social grouping, social needs of infants, structure and substrate, foraging opportunities and manipulanda) as well as stimulation of all five senses, various food treats and daily interaction with animal care operations staff and/or investigators.

The anterior lamina cribrosa (LC) and ONH were cross-sectionally examined *in vivo* at a single time-point in left and right eyes of 6 normal rhesus monkeys (*Macaca mulatta*) with a mean age of 2.8 ± 0.9 years. Normal animals in this study were labeled as M0##. In addition, longitudinal examination of the anterior LC and ONH was performed *in vivo* in fellow eyes of 7 rhesus monkeys induced with unilateral experimental glaucoma (EG). EG eyes were labeled as OHT-##. Argon laser treatment of the trabecular meshwork was used to elevate the intraocular pressure of each monkey’s right eye, while fellow eyes served as controls [39–42]. In brief, monkeys were anesthetized with ketamine (20–30 mg/kg) and xylazine (0.8–0.9 mg/kg) and a topical corneal anesthetic (0.5% proparacaine) was instilled in the treated eye. A clinical Argon laser was used to deliver treatment burns over 180 degrees of the mid-trabecular meshwork in each session using a single mirror gonioprism. To avoid large IOP spikes, multiple treatments (1–7, minimally separated by 3 weeks) were used to slowly build up and create sustained pressures of 30–50 mm Hg. Although the experimental procedure involved a laser treatment, the procedure is similar to the Argon laser trabeculoplasty that is used as a common surgical treatment for patients with glaucoma, which is not painful. The mean (± SD) age of the 7 EG monkeys at the time-point of the first laser treatment was 3.7 ± 0.5 years.

Monkeys were anesthetized with 20–25 mg/kg ketamine and 0.8–0.9 mg/kg xylazine to minimize eye movements during *in vivo* imaging [43]. Atropine sulfate (0.04 mg/kg) was added to reduce the effects of xylazine on the heart rate and each monkey’s pupils were dilated with 2.5% phenylephrine and 1% tropicamide. A pharmacological agent (IOPIDINE, Alcon Laboratories, Inc., Fort Worth, TX, USA or COMBIGAN, Allergan, Inc., Irvine, CA, USA) was used at the start of each imaging experiment to reduce IOPs to levels at or near baseline and ensure that the structural changes measured during the experiment were due to chronic changes resulting from a sustained elevation in IOP (and not a transient pressure spike). IOP was measured using an applanation tonometer (Tono-Pen XL Applanation Tonometer; Reichert, Inc., NY, USA). Maximum IOP and cumulative IOP difference were monitored in control and EG eyes after the first laser procedure. Cumulative IOP difference was calculated at each time-point by successively integrating IOP up to the given time-point in the EG eye and subtracting the corresponding cumulative IOP from the fellow control eye [44]. A head mount was used to stabilize the monkey’s head during spectral domain optical coherence tomography (SDOCT) and AOSLO imaging. A lid speculum was used to keep the eyelids open and a contact lens was placed on the monkey eye to prevent corneal dehydration during imaging. Each monkey had at least one baseline imaging session prior to the first laser procedure and was subsequently imaged at time-points spaced approximately every 2 weeks following the first laser session.

### Biometric measurements and image scaling

An ocular biometer (IOLMaster; Carl Zeiss Meditec, AG, Jena, Germany) was used to measure axial length, anterior chamber depth, and anterior corneal curvature in all eyes at every time-point. These biometric measurements were used to laterally scale SDOCT and AOSLO images using previously defined methods [34].

### Spectral domain optical coherence tomography (SDOCT) imaging and analysis

**Fig 1:**
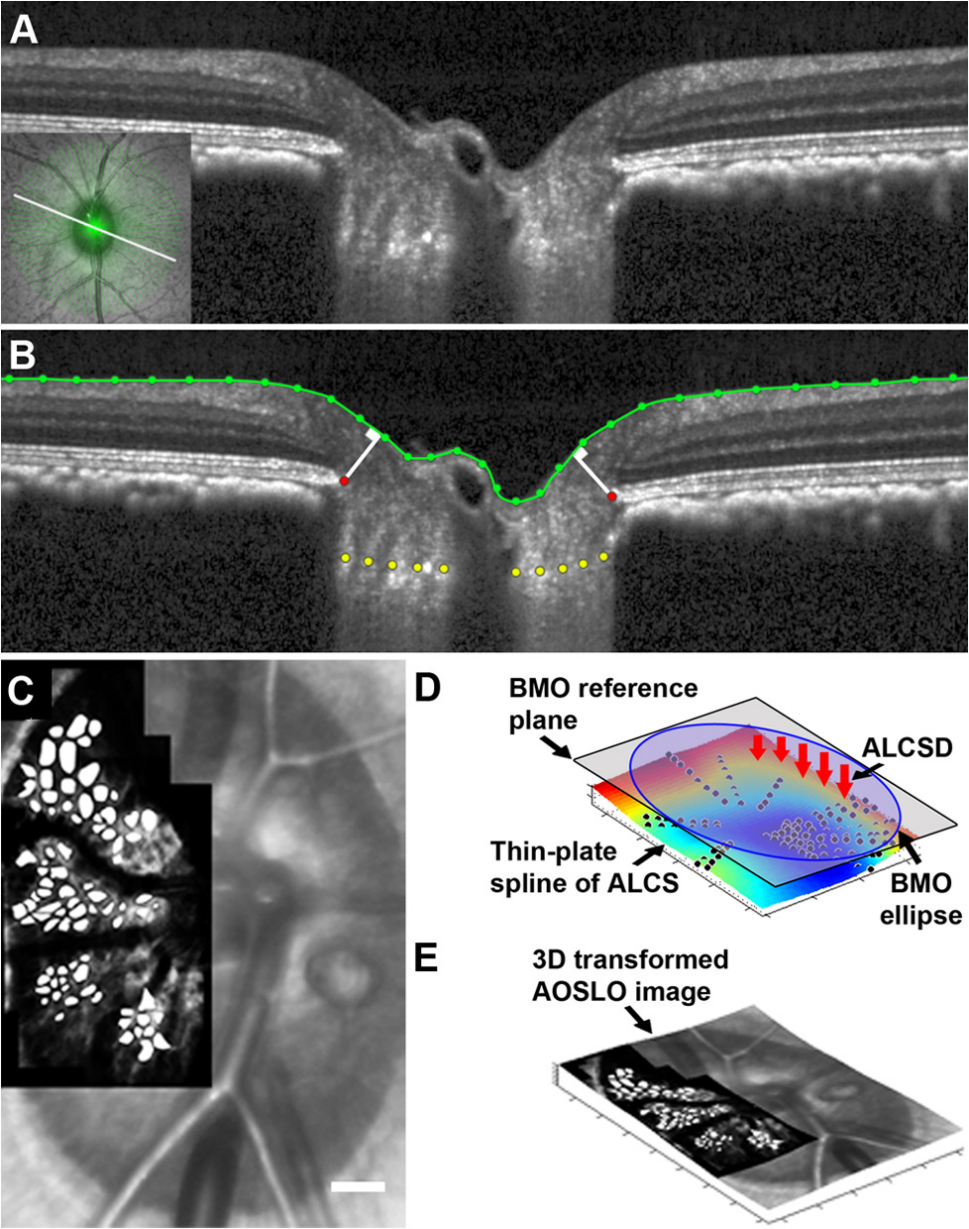
Semi-automated marking of ONH features and ALCS pores from SDOCT and AOSLO images. (A) A single SDOCT radial B-scan of the ONH and LC acquired from the left eye of a normal monkey, M067. The scan location is denoted by the bold white line in the inset (lower left corner) which shows the corresponding *en face* SLO image of the ONH and location of all radial B-scans (green lines) acquired in the same eye. (B) The same B-scan as in (A) with manually marked ONH features, including the termination points of the RPE/BM interface (red dots) and ALCS (yellow dots), and the semi-automatically segmented ILM (green dots and line). The minimum distance from each termination point of the RPE/BM interface to the ILM was calculated on each side of the neural canal opening (white lines) in each B-scan. Mean MRW was computed as the average of all minimum widths across all B-scans. (C) A 2D AOSLO image of the ALCS overlaid on the SLO image from the same normal eye (M067) with manually marked LC pores in white. Scale bar: 150 μm. (D) A thin-plate spline surface was fit in 3D to the marked ALCS points (black dots that lie above and below the fitted surface) and used to calculate mean ALCSD (red arrows) from the BMO plane (gray). (E) 3D transformed AOSLO image of the ALCS following registration and projection of the 2D AOSLO image from (C) onto the 3D thin-plate spline surface from (D). Pores were quantified from the 3D transformed images to better represent the physiological LC surface geometry.

Scanning laser ophthalmoscope (SLO) images of the ONH (30° x 30°) were acquired (Spectralis HRA+OCT; Heidelberg Engineering, Heidelberg, Germany) at a single time-point in bilaterally normal monkey eyes and at all longitudinal time-points (approximately every 2 weeks) in EG monkey eyes. Mean peripapillary RNFL thickness (RNFLT) was measured in each EG monkey from 12° circular scans centered on the ONH using the instrument’s software at every time-point. Cross-sectional radial scans (20° field, 48 B-scans) centered on the ONH were acquired in each normal and EG monkey using the same SDOCT instrument at every time-point. Scans from follow-up time-points in EG monkeys were registered to the baseline time-point using the SDOCT instrument’s software to ensure the same region was imaged every time. The termination points of the retinal pigment epithelium (RPE)/Bruch’s membrane (BM) interface and the anterior lamina cribrosa surface (ALCS) were manually marked in as many B-scans as possible using a custom program (MATLAB; The MathWorks, Inc., Natick, MA) (Fig 1A and 1B). Mean anterior lamina cribrosa surface depth (ALCSD) was computed as the mean distance between a plane best-fit to the marked Bruch’s membrane opening (BMO) points and a thin-plate spline surface that was fit to the marked ALCS points to model the ALCS in three dimensions (Fig 1D) [37]. The ALCS was further characterized by computing its mean radius of curvature (RoC) within the projection of the BMO ellipse (an ellipse best-fit to the marked BMO points) onto the thin-plate spline model of the ALCS [37]. In addition, the internal limiting membrane (ILM) was automatically segmented using the SDOCT instrument’s software and manually corrected to determine mean minimum rim width (MRW), the minimum distance between each BMO point and the ILM averaged across all B-scans (Fig 1A and 1B). Mean ALCSD, RoC, MRW, and RNFLT were quantified at every time-point in fellow eyes of all EG monkeys. While ONH parameters were quantified in the EG eye of monkey OHT-65 at every time-point, it was not possible to quantify ONH parameters in the control eye of the same monkey due to poor image quality resulting from the eye’s poor optics.

### Adaptive optics scanning laser ophthalmoscope (AOSLO) imaging and analysis

The methods for imaging the ALCS microarchitecture using an AOSLO have been described previously [34]. Briefly, adaptive optics reflectance imaging of the plane of best-focus of the ALCS was performed throughout as much of the optic nerve head as possible. Videos were acquired over a 1.5° field at a rate of 25 Hz using an 840 nm superluminescent diode (S-Series Broadlighter, Superlum, Carrigtwohill, Ireland) [34,37,45]. Registered images were created from AOSLO videos using a custom program (MATLAB; The MathWorks, Inc., Natick, MA) and combined to generate a montage of the ALCS microarchitecture (Adobe Photoshop; Adobe Systems, San Jose, CA) in fellow eyes of normal monkeys and in the control and EG eyes of each EG monkey at every time-point.

Local image contrast in each ALCS montage was further improved using a contrast limiting adaptive histogram equalization (CLAHE) algorithm (ImageJ; developed by Wayne Rasband, National Institutes of Health, Bethesda, MD; available at http://rsb.info.nih.gov/ij/index.html) and pore boundaries were manually marked (Adobe Photoshop) using previously described methods found to have excellent repeatability and reproducibility (Fig 1C) [34]. Because AOSLO images of the ALCS represent a projected view of the 3D LC surface, our 2D AOSLO images were transformed into their approximate 3D configuration by registering and projecting the AOSLO image onto the thin-plate spline representation of the ALCS (Fig 1D) [37]. Following 3D transformation (Fig 1E), the area, elongation, and nearest neighbor distance (NND) of all marked pores were quantified in each montage and analyzed globally, regionally (centrally vs. peripherally) and within 60° sectors. Pores were separated into central and peripheral regions by dividing the area contained within the BMO ellipse into 2 regions of equal area. Meridians were also constructed at 60° intervals from the major axis of the same BMO ellipse in order to locally examine pores in superotemporal, temporal, and inferotemporal sectors. Pore parameters were compared longitudinally throughout the progression of experimental glaucoma. Again, due to the poor image quality resulting from its poor optical quality, ALCS pore geometry was not quantified in the control eye of monkey OHT-65 at any time-point. However, AOSLO images of the ALCS in monkey OHT-65’s EG eye were acquired and analyzed at every time-point.

### Statistical analysis

All statistical tests (with the exception of the subsequently described Moran’s I calculations) were performed using a commercially available platform (SigmaPlot; Systat Software Inc., San Jose, CA). A paired t-test was used to compare mean ALCSD, mean RoC, and mean MRW between fellow eyes of all normal monkeys. The non-parametric Mann-Whitney rank sum test was used to compare and assess statistically significant differences in LC pore parameters within individual eyes and between fellow eyes of normal monkeys. For EG monkeys, the coefficients of repeatability [1.96 **x** SD **x** $\sqrt{2}$] and variation [(SD/mean) **x** 100] were calculated for mean ALCSD, mean RoC, mean MRW, and RNFLT across all time-points in control eyes. The 95% confidence intervals were subsequently calculated for each ONH parameter in each control eye. The first time-point of significant change for each ONH parameter in each EG eye was determined as the first time-point that fell outside the 95% confidence interval (established from the control eyes) and had no subsequent time-points with values that fell back within the confidence interval. To determine the first significant time-point of change in each ONH parameter in monkey OHT-65 (the monkey with poor image quality resulting from poor optics in the control eye), data from the EG eye of OHT-65 was compared to the 95% confidence interval generated from all control eye data in the other 6 monkeys. The first time-point that fell outside the 95% confidence interval that was generated from all other control eyes (and had no later time-points with values within the confidence interval) represented the first significant time-point of change for ONH parameters in the EG eye of OHT-65.

Mean pore area, elongation, and NND were compared globally, regionally (centrally and peripherally) and by sector (superotemporally, temporally, and inferotemporally) in fellow eyes of all normal monkeys, and regionally and by sector in fellow eyes of all EG monkeys at all time-points. 95% confidence intervals were calculated for pore area, elongation, and NND in all EG eyes across early time-points that showed no statistically significant differences in all pore parameters as determined using a Mann-Whitney rank sum test. *P* values >.05 were taken to represent no statistically significant differences from baseline. The first significant time-point of change in a pore parameter was determined to be the first time-point that fell outside the 95% confidence interval. Statistical comparisons of pore parameters between follow-up time-points and baseline time-points within a given EG eye were conducted only for sectors or regions that were present at the given follow-up time-point and at baseline time-points. Finally, to investigate the temporal sequence of structural change in RNFLT, ONH parameters, and ALCS microarchitecture, event-based Kaplan-Meier curves were generated and a log-rank test was performed to test statistically significant differences between the Kaplan-Meier curves constructed for each parameter (mean ALCSD, mean MRW, ALCS pore geometry, mean RoC, and mean RNFLT) across all EG eyes.

Cluster analysis and the Moran’s I spatial autocorrelation statistical test were performed in all normal monkeys to compare the spatial arrangement of marked pores within each eye. Pores were clustered into 1 of 3 bin sizes (small, medium, or large) in each eye depending on the magnitude of the parameter being evaluated (e.g., pore area) (Orange; Bioinformatics Laboratory, University of Ljubljana, Slovenia) [46]. The selection of three bins allowed for an optimal separation of the data and best ensured that neither too many pores were grouped into too few bins nor that too few pores were grouped into too many bins. The range of values contained within each bin was determined such that the mean value of the analyzed parameter within a given bin (e.g., large area pores) was maximally separated from the mean value of the next nearest bin (e.g., medium area pores). A Moran’s I statistical test was then performed on all marked pores in each eye (R; Institute for Digital Research and Education, University of California Los Angeles, CA) to determine whether a statistically significant local spatial pattern existed for pores based on the values of the examined parameter (e.g., pore area). For example, when examining pore area, pores that were colocated with pores of very similar area would have a high positive correlation (+1 indicates perfect spatial correlation; e.g., all large pores clustered in one region/sector and all small pores clustered in a different region/sector), whereas pores that were adjacent to pores of dissimilar area would result in a more negative Moran’s I index value, suggesting increased spatial dispersion (-1 indicates perfect spatial dispersion; e.g., periodic alternation of large, medium and small sized pores throughout the ALCS) [47]. A Moran’s I index value near 0 indicates a random spatial arrangement of pore areas where there is no discernable clustering. The cluster analysis and Moran’s I test were performed to investigate the spatial arrangement of pores based on pore area, elongation, and NND. A *P* value was calculated to determine the statistical significance of the Moran’s I index value. *P* values < .05 were taken to represent a significant spatial autocorrelation in pores based on the pore parameter analyzed.

## Results

### Structural comparisons between fellow, normal eyes

**Fig 2:**
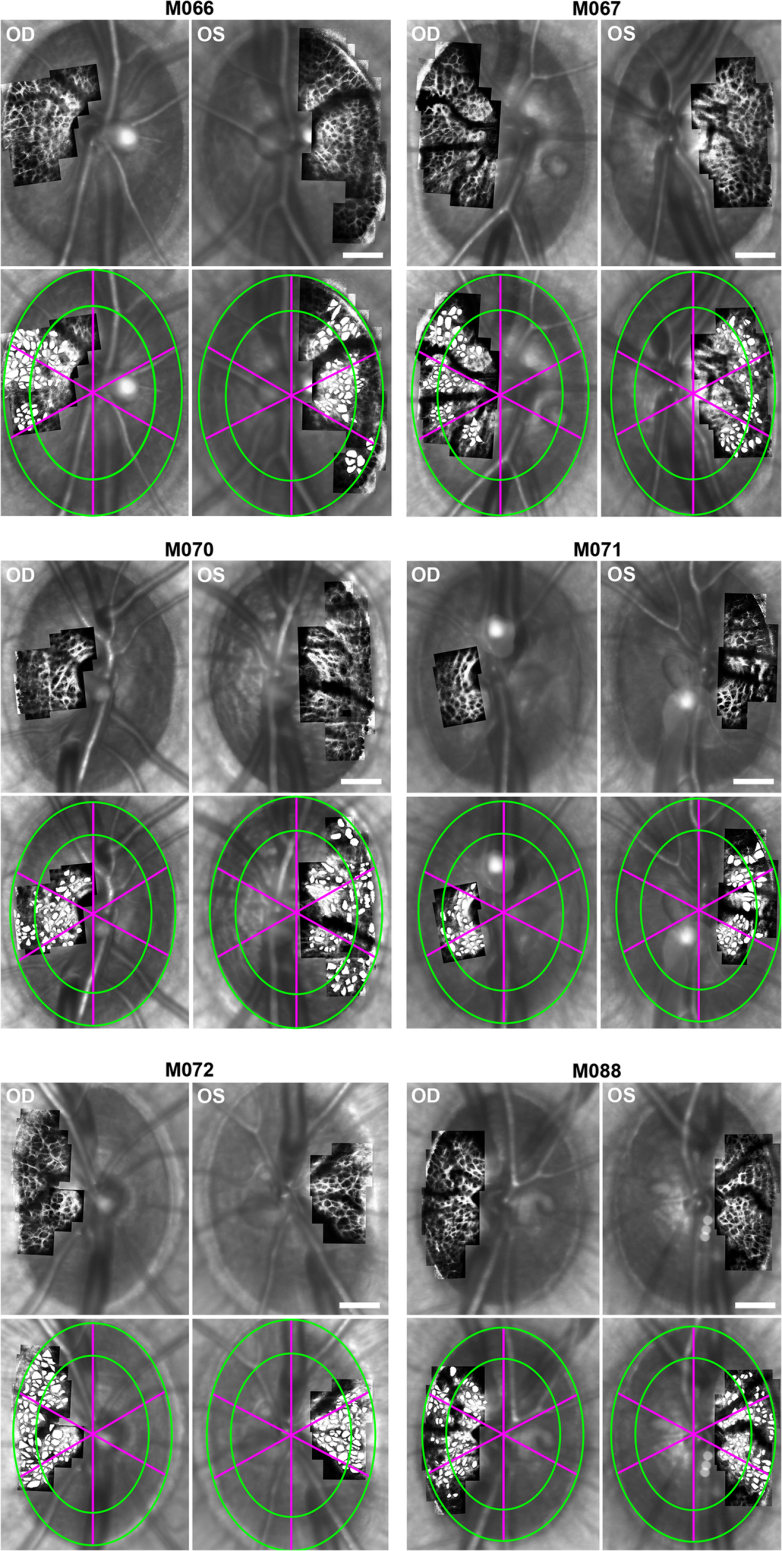
LC pore parameters were analyzed on global and local levels in all normal eyes. (Rows 1, 3, 5) AOSLO montages of the ALCS scaled, registered, and overlaid on the corresponding SLO images in right and left eyes of 6 normal monkeys. (Rows 2, 4, 6) After manually marking LC pores (filled in white), pores were examined globally, in central and peripheral regions (separated by green boundaries) and in 60° sectors (divided by fuchsia meridians). Scale bar: 100 μm.

Optic nerve head and ALCS pore parameters were similar between fellow eyes of bilaterally normal monkeys. Paired t-tests revealed no statistically significant differences (*P*>.05) in mean ALCSD, mean RoC, and mean MRW values calculated from marked SDOCT radial B-scans between left and right eyes across all 6 normal monkeys (S1 Fig and Table 1). High-resolution AOSLO images of the temporal side of the ALCS were acquired and overlaid on the corresponding SLO images in fellow eyes of all normal monkeys (Fig 2, rows 1, 3, and 5). The mean percentage of the ALCS microarchitecture that was imaged within the BMO ellipse was 25.4 ± 7.1% across all normal eyes. The pores that were manually marked in each montage are shown in the images directly below each raw AOSLO/SLO image (Fig 2, rows 2, 4, and 6). The mean number of pores analyzed across all normal eyes was 77 ± 12. Mean (± st. dev.) values of ALCS pore area, elongation, and NND quantified globally, regionally and by sector are shown in Table 2 across all right eyes and left eyes. Globally, statistically significant differences in LC pore parameters were found between fellow eyes in two monkeys (M070 and M072) for pore area, in no monkeys for pore elongation, and in three monkeys (M066, M070, and M072) for pore NND (*P* < .05). In addition to global comparisons of pores in normal monkeys, pores were segmented into regions of equal area (central and peripheral) and into 60° sectors in all eyes in order to better compare ALCS pore parameters on a local level within eyes (Fig 2, rows 2, 4, 6). Statistically significant differences were found between central and peripheral regions in only 2 of 12 eyes (M070, OS; M071, OS) (Mann-Whitney rank sum test, *P* < .05). Pore elongation was not significantly different between central and peripheral regions in all eyes while pore NND was significantly larger in the peripheral region in 2 eyes (M071, OS; M088, OD). On a sector level, pore area was significantly larger in the superotemporal and/or inferotemporal sectors compared to the temporal sector in 4 eyes (M066, OS; M067, OD; M072, OS; M088, OD). Pore NND was also significantly larger in the superotemporal and/or inferotemporal sectors compared to the temporal sector in 6 eyes (M066, OS; M067, OD; M070, OD; M071, OD; M072, OS; M088, OS), while pore elongation was significantly smaller in the superotemporal and inferotemporal sectors compared to the temporal sector in 3 eyes (M070, OS; M071, OD and OS).

**Table 1:** ONH parameters in fellow eyes of 6 bilaterally normal monkeys and mean inter-eye differences.

	Mean ALCSD (μm)		Mean RoC (mm)		Mean MRW (μm)	
Monkey	OD	OS	OD	OS	OD	OS
M066	191.6 ± 36.2	202.4 ± 29.0	3.4	4.1	281.8 ± 49.8	284.5 ± 48.3
M067	175.5 ± 32.7	193.2 ± 23.0	4.2	3.3	242.8 ± 54.7	238.4 ± 45.0
M070	177.8 ± 30.9	195.2 ± 39.0	3.0	3.0	232.7 ± 58.5	222.0 ± 57.2
M071	202.0 ± 32.3	206.4 ± 24.1	3.6	2.7	249.9 ± 55.3	257.1 ± 65.2
M072	199.3 ± 53.5	190.0 ± 41.8	3.7	3.1	299.1 ± 45.5	301.3 ± 66.7
M088	206.6 ± 34.6	206.1 ± 25.6	4.0	3.9	283.7 ± 55.2	263.8 ± 53.3
Mean (across monkeys)	192.1 ± 13.0	198.9 ± 7.0	3.6 ± 0.4	3.4 ± 0.5	265.0 ± 26.7	261.2 ± 29.1
Mean Absolute Inter-eye Difference	10.0 ± 6.9 μm (5.3 ± 3.8%)		0.5 ± 0.4 mm (15.0 ± 11.0%)		7.9 ± 6.7 μm (3.1 ± 2.5%)	

**Table 2:** Mean (± st. dev.) pore parameters across all eyes of 6 bilaterally normal monkeys on global, regional, and sector scales.

	Mean Pore Area (μm^2^)		Mean Pore Elongation		Mean Pore NND (μm)	
	OD	OS	OD	OS	OD	OS
Global	1023 ± 201	1120 ± 227	1.65 ± 0.04	1.68 ± 0.05	41.8 ± 3.6	41.4 ± 4.5
Central	971 ± 187	1032 ± 136	1.64 ± 0.07	1.66 ± 0.05	40.0 ± 2.5	40.6 ± 4.4
Peripheral	1122 ± 261	1315 ± 587	1.65 ± 0.10	1.74 ± 0.10	39.3 ± 4.1	44.1 ± 7.2
Superotemporal	1179 ± 239	1122 ± 312	1.67 ± 0.09	1.66 ± 0.05	46.4 ± 8.6	43.9 ± 6.0
Temporal	932 ± 250	1147 ± 348	1.70 ± 0.06	1.76 ± 0.11	38.1 ± 3.0	40.4 ± 4.4
Inferotemporal	964 ± 169	1229 ± 428	1.55 ± 0.12	1.55 ± 0.08	39.6 ± 1.8	42.6 ± 6.8

A cluster analysis was performed in fellow eyes of each normal monkey to examine the spatial arrangement of ALCS pores based on their area, elongation and NND. (Representative examples from 3 monkey eyes are shown in S2 Fig to illustrate the variability in the distribution of ALCS pore areas.) Significant positive spatial autocorrelations (*P* < .05) in pore area, elongation and NND were found in 6 of 12, 7 of 12 and 11 of 12 eyes, respectively (S1 Table). These findings indicate that pores of similar area (e.g., M067 in panel C of S2 Fig), shape (elongation) or spacing (NND) tended to be more spatially clustered in these eyes.

### Longitudinal structural changes in EG

**Fig 3:**
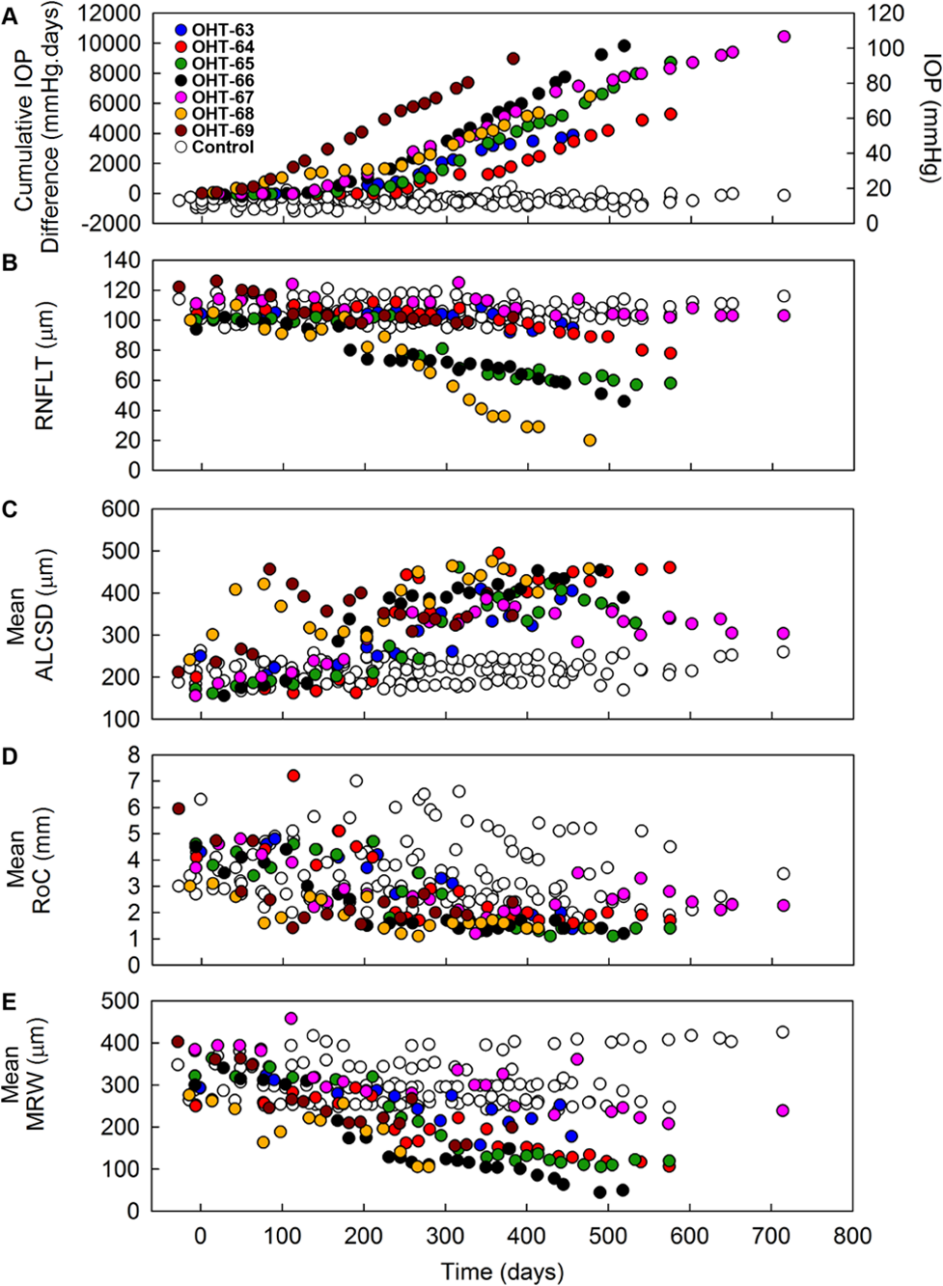
Longitudinal changes in IOP, RNFLT, and ONH parameters in control eyes and eyes with experimental glaucoma. Plots of (A) cumulative IOP difference, (B) RNFLT, (C) mean ALCSD, (D) mean RoC, and (E) mean MRW as a function of study time for all monkeys. The legend presented in panel (A) denotes which filled colored circles correspond to each EG eye and is also applicable to panels (B)–(E). Open white circles represent control eyes. (In [A], open white circles represent control IOP data corresponding to the right-most axis). Progressive increases were measured in cumulative IOP difference and mean ALCSD with increasing study duration, whereas progressive decreases were measured in RNFLT, mean RoC, and mean MRW in nearly all EG eyes.

Intraocular pressure (IOP) data measured in the control and EG eyes of 7 EG monkeys are shown in Table 3. Mean values of unaltered IOP (± SD) across all time-points were 13.2 ± 1.4 mmHg in control eyes and 27.9 ± 4.2 mmHg in EG eyes. On average, the maximum unaltered IOP was 17.9 ± 1.7 mmHg across control eyes and 50.4 ± 4.8 mmHg across EG eyes. IOP was pharmacologically lowered to near baseline levels at the start of each imaging experiment. Across all EG eyes, the mean pharmacologically-reduced IOP was 19.3 ± 2.5 mmHg, which was 6.0 mmHg larger than the mean unaltered IOP in the fellow control eyes. Over a mean experiment duration of 528 ± 107 days, the mean cumulative IOP difference was 7,654 ± 2,472 mmHg-days across all 7 monkeys. A steady increase in cumulative IOP difference was observed for each monkey (Fig 3A).

**Table 3:** IOP data for control and EG eyes throughout the duration of the study.

		Mean IOP (± SD) (mmHg)		Maximum IOP (mmHg)			
Animal ID	Age (yrs)	Control Eye	EG Eye	Control Eye	EG Eye	Reduced IOP in EG Eye (mmHg)	Cumulative IOP difference (mmHg-days)
OHT-63	3.9	14.1 ± 1.1	24.1 ± 12.1	16	49	18.4 ± 5.0	3880
OHT-64	4.2	14.5 ± 2.1	25.5 ± 12.7	21	47	18.3 ± 6.4	5280
OHT-65	3.0	10.6 ± 2.6	25.4 ± 14.6	17	51	17.2 ± 9.0	8720
OHT-66	3.2	14.5 ± 2.0	33.3 ± 15.5	19	60	18.2 ± 7.8	9824
OHT-67	3.3	12.7 ± 2.6	26.8 ± 12.4	18	51	18.1 ± 7.7	10441
OHT-68	3.9	13.1 ± 2.5	25.9 ± 9.9	17	45	20.0 ± 9.6	6461
OHT-69	4.2	13.2 ± 1.7	34.5 ± 11.3	17	50	24.7 ± 10.4	8975
Mean ± SD	3.7 ± 0.5	13.2 ± 1.4	27.9 ± 4.2	17.9 ± 1.7	50.4 ± 4.8	19.3 ± 2.5	7654 ± 2472

Retinal nerve fiber layer thickness (RNFLT) and ONH parameters remained stable in control eyes of EG monkeys for the duration of the study. Mean (± SD) RNFLT, ALCSD, RoC, and MRW values calculated across all control eyes were 105.6 ± 6.8 μm, 214.0 ± 25.3 μm, 3.6 ± 1.0 mm, and 308.7 ± 55.1 μm, respectively (Table 4). Coefficients of variation and repeatability were small for these parameters in all control eyes (Table 4), indicating low variability and high precision in measured values over time.

**Table 4:** RNFLT and mean ONH parameters averaged across all study time-points for each control eye.

	RNFLT			Mean ALCSD			Mean RoC			Mean MRW		
Animal ID	Mean ± SD (μm)	Coeff. of Repeat. (μm)	Coeff. of Variation (%)	Mean ± SD (μm)	Coeff. of Repeat. (μm)	Coeff. of Variation (%)	Mean ± SD (μm)	Coeff. of Repeat. (μm)	Coeff. of Variation (%)	Mean ± SD (μm)	Coeff. of Repeat. (μm)	Coeff. of Variation (%)
OHT-63	101.9±1.3	3.5	1.3	225.9±14.5	40.1	6.4	3.9±1.1	3.2	29.5	299.7±5.8	15.9	1.9
OHT-64	104.0±1.5	4.1	1.4	215.1±3.9	10.8	1.8	5.1±0.9	2.4	17.4	252.1±5.3	14.7	2.1
OHT-65	106.8±1.9	5.2	1.7	—	—	—	—	—	—	—	—	—
OHT-66	97.3±1.9	5.4	2.0	184.5±5.9	16.5	3.2	2.6±0.3	0.9	11.9	297.3±6.5	18.1	2.2
OHT-67	111.8±2.0	5.5	1.8	237.8±14.3	39.6	6.0	2.7±0.8	2.3	31.3	398.2±13.5	37.4	3.4
OHT-68	100.5±2.0	5.7	2.0	238.7±9.5	26.4	4.0	3.0±0.5	1.4	17.1	259.7±6.1	17.0	2.4
OHT-69	116.9±1.8	4.9	1.5	182.1±10.5	29.2	5.8	4.3±1.0	2.8	23.2	345.4±6.2	17.0	1.8
Mean ± SD	105.6±6.8	4.9±0.8	1.7±0.3	214.0±25.3	27.1±11.9	4.5±1.8	3.6±1.0	2.2±0.9	21.7±7.6	308.7±55.1	20.0±8.6	2.3±0.6

‘—’—ONH parameters were not quantified in the control eye of OHT-65.

Significant longitudinal changes were measured in RNFLT and ONH parameters (mean ALCSD, mean RoC, and mean MRW) in all EG eyes except for monkey OHT-67, who had no significant changes in mean RoC (3.7 mm at baseline and 2.3 mm at the final time-point) and RNFLT (111 μm at baseline and 103 μm at the final time-point). A progressive increase in mean ALCSD was measured in all EG eyes, whereas RNFLT, mean RoC, and mean MRW tended to decrease throughout the study (Fig 3B–3E). On average across all EG eyes, the maximum value of mean ALCSD (448.1 ± 37.7 μm) and minimum values of RNFLT (70.3 ± 30.4 μm), mean RoC (1.3 ± 0.2 mm) and mean MRW (125.7 ± 52.3 μm) represented a +133%, -34%, -70%, and -61% average change from baseline values (Table 5).

**Table 5:** RNFLT and mean ONH parameters averaged across all study time-points for each EG eye.

	RNFLT		Mean ALCSD		Mean RoC		Mean MRW	
Animal ID	Mean ± SD (μm)	Minimum (μm)	Mean ± SD (μm)	Maximum (μm)	Mean ± SD (mm)	Minimum (mm)	Mean ± SD (μm)	Minimum (μm)
OHT-63	102.4 ± 4.8	92	293.5 ± 66.4	409.6	3.1 ± 1.2	1.3	252.6 ± 47.0	156.9
OHT-64	100.3 ± 10.1	78	348.3 ± 121.0	494.5	2.8 ± 1.5	1.5	188.7 ± 61.1	106.1
OHT-65	78.8 ± 18.7	57	296.6 ± 94.2	460.7	2.6 ± 1.4	1.1	208.6 ± 96.5	105.2
OHT-66	75.5 ± 15.9	46	340.2 ± 102.9	454.6	2.2 ± 1.1	1.2	162.2 ± 94.2	44.2
OHT-67	109.2 ± 6.5	101	295.5 ± 66.1	385.7	2.8 ± 0.9	1.2	306.4 ± 67.2	207.5
OHT-68	69.8 ± 29.3	20	385.1 ± 69.1	475.0	1.9 ± 0.6	1.1	196.9 ± 56.5	104.8
OHT-69	106.5 ± 9.4	98	337.6 ± 64.4	456.7	2.6 ± 1.2	1.4	256.8 ± 74.6	155.1
Mean ± SD	91.8 ± 16.4	70.3 ± 30.4	328.1 ± 34.5	448.1 ± 37.7	2.6 ± 0.4	1.3 ± 0.2	224.7 ± 49.5	125.7 ± 52.3

**Fig 4:**
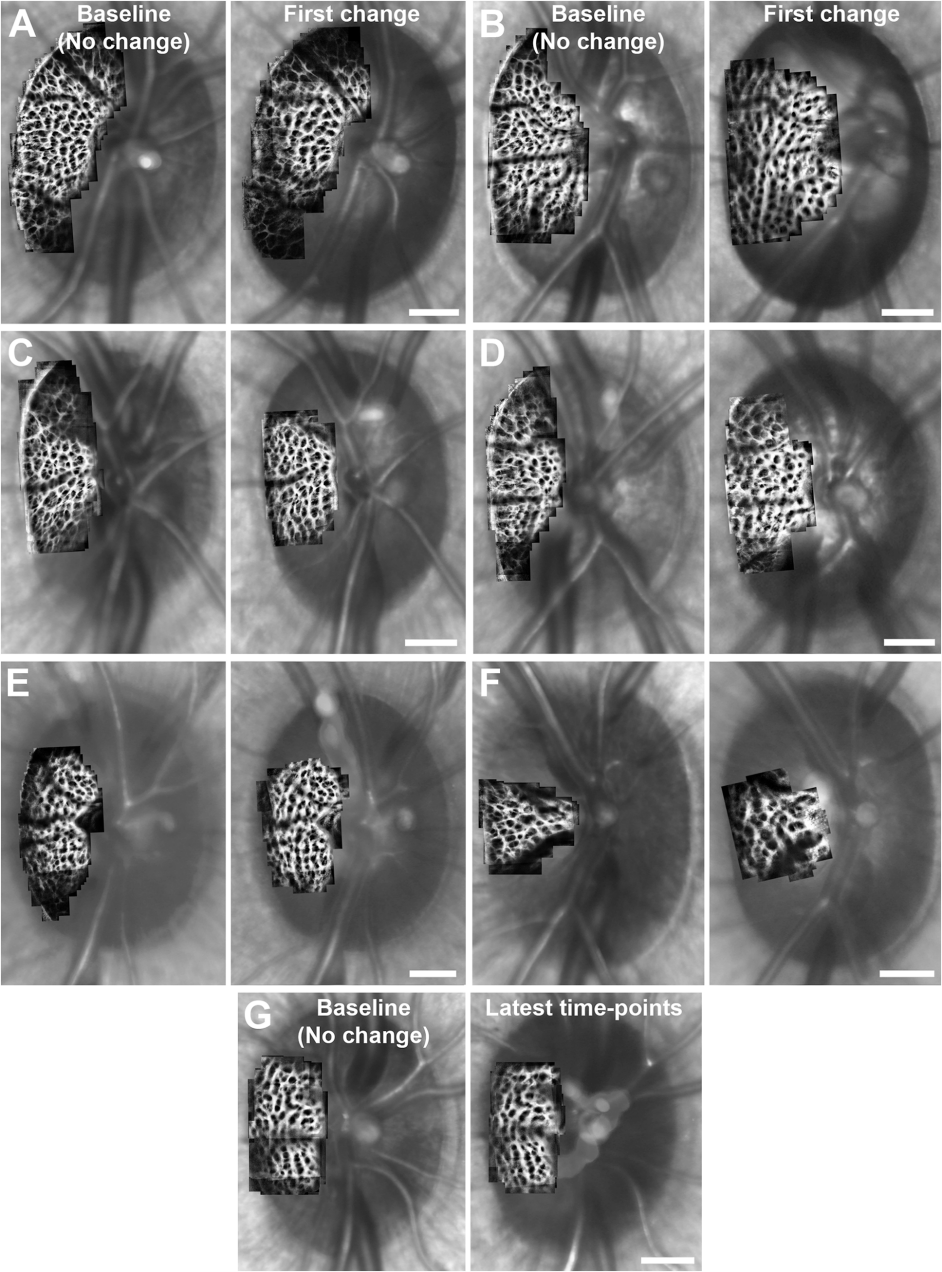
Distinct changes in LC beam and pore structure were observed in early EG. (A-F) AOSLO montages of the ALCS were constructed, registered, and averaged across multiple time-points in the same EG eye for each monkey and overlaid on the corresponding SLO images to show the LC before (left) and after (right) the first statistically significant changes were seen in ALCS pore geometry in early experimental glaucoma. Large differences in beam and pore structure can be seen in 6 of 7 EG eyes over time. (A: OHT-63; B: OHT-64; C: OHT-65; D: OHT-66; E: OHT-68; F: OHT-69.) (G) ALCS pore structure did not change significantly over time in the EG eye of monkey OHT-67. Scale bar: 300 μm.

While ALCS pore geometry did not significantly change over time in any control eye (*P*>.05; see S3 Fig for an example), distinct structural changes in ALCS beams and pores were observed over time in all EG eyes but one (OHT-67). (No significant changes were measured in ALCS pore geometry, mean RoC and RNFLT in the EG eye of OHT-67 throughout the study.) Fig 4 shows AOSLO montages of ALCS microarchitecture at baseline time-points (columns 1 and 3) and time-points corresponding to the first statistically significantly measured change in ALCS pore geometry (columns 2 and 4) in each EG eye. To better visualize the consistency of pore geometry prior to the first measured change, each baseline montage represents an image averaged from baseline time-points and time-points that had no measured differences in ALCS pore geometry compared to baseline. Each “first change” montage represents an image averaged from the time-point of first significant change in ALCS pore geometry and any subsequent time-points that showed no statistically significant change in ALCS pore geometry from the first time of measured change. The mean percentage of the ALCS microarchitecture that was imaged within the BMO ellipse throughout the entire course of EG was 24.3 ± 4.3% across all EG eyes. Table 6 summarizes mean ALCS pore geometry at the first time-point of change in LC pores compared to baseline and earlier time-points of no change across all EG eyes. The mean numbers of pores analyzed across all control and EG eyes were 72 ± 7 and 71 ± 15, respectively. Mean ALCS pore area significantly increased centrally, peripherally, and in the temporal sector at the time of first change. Correspondingly, mean ALCS pore NND significantly increased in all regions and sectors (except inferotemporally) at the same time. However, mean ALCS pore elongation did not significantly change on local scales across all EG eyes at the time of first change in pore area and NND (*P>*.05).

**Table 6:** Values of mean (± SD) pore parameters measured at baseline (No change) and at the first time-point of statistically significant change in LC pore geometry (First change) on regional (central, peripheral) and sector scales.

	Area (μm^2^)		Elongation		NND (μm)	
	Baseline (No change)	First change	Baseline (No change)	First change	Baseline (No change)	First change
Central	801.9 ± 89.0	982.4 ± 146.4*	1.72 ± 0.08	1.71 ± 0.09	36.1 ± 2.6	40.3 ± 6.2*
Peripheral	920.6 ± 277.9	1301.8 ± 252.4*	1.77 ± 0.16	1.93 ± 0.46	39.5 ± 3.5	47.8 ± 7.3*
Superotemporal	919.2 ± 171.0	1141.1 ± 264.4	1.69 ± 0.12	1.72 ± 0.08	42.0 ± 10.1	43.3 ± 5.8*
Temporal	823.3 ± 83.4	1115.0 ± 162.1*	1.81 ± 0.13	1.83 ± 0.12	36.6 ± 2.6	42.9 ± 6.3*
Inferotemporal	961.9 ± 280.9	1049.6 ± 454.7	1.63 ± 0.08	1.70 ± 0.23	40.4 ± 3.8	40.5 ± 7.9

‘*’—Statistically significant difference in a LC pore parameter compared to previous baseline time-points with no change (*P* < .05).

**Fig 5:**
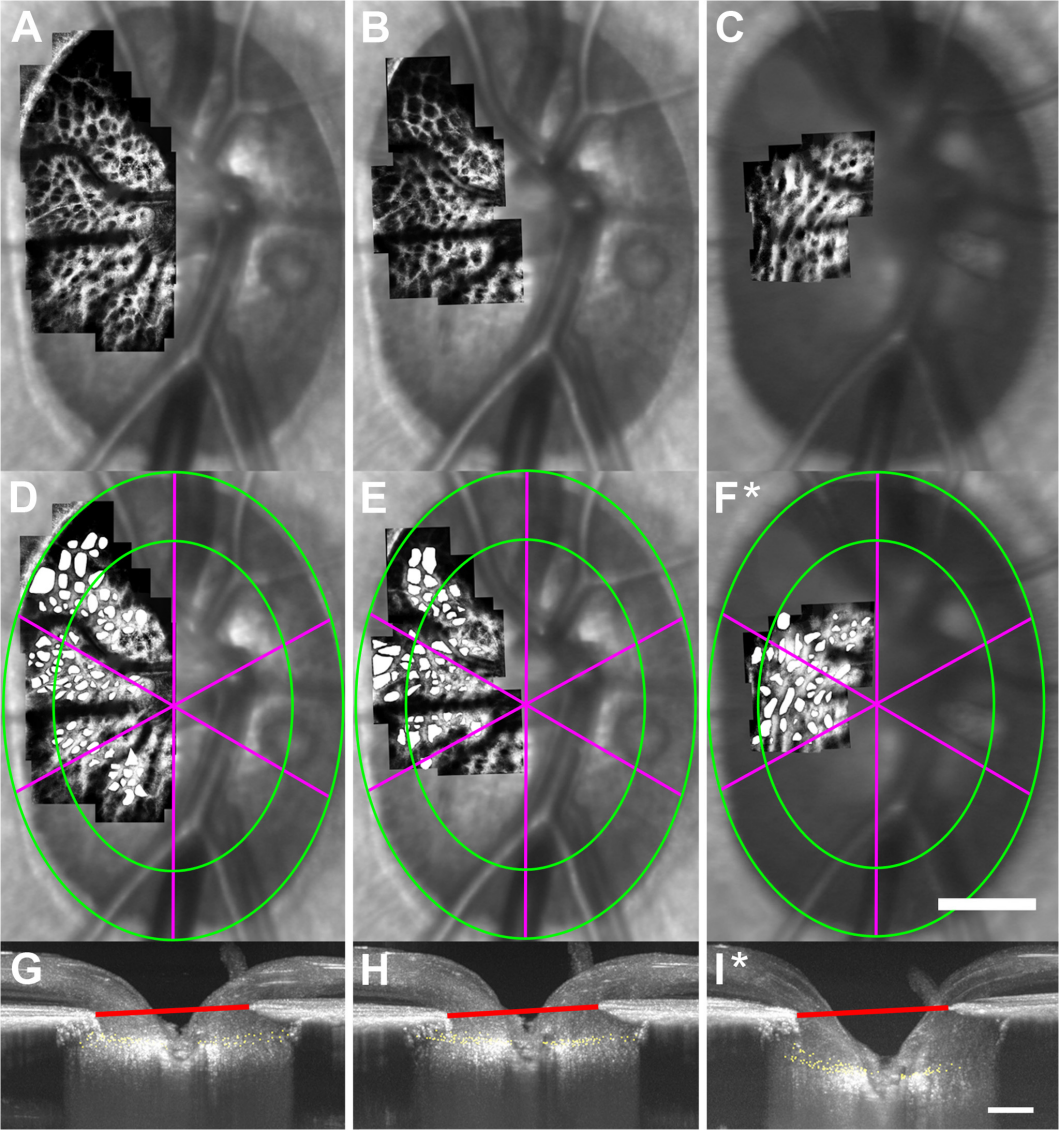
The first significant changes in ALCS pore geometry (relative to baseline time-points) occurred simultaneously with the first significant change measured in mean ALCSD in the EG eye of monkey OHT-64. Images of (A-C) ALCS microarchitecture and (G-I) the ONH were each acquired at baseline (left column), a representative follow-up time-point (middle column, 210 days after the initial laser treatment) that showed no significant change in ALCS pore geometry or mean ALCSD, and the time-point corresponding to the first significant change (*) in ALCS pore geometry and mean ALCSD (right-most column, 238 days after the initial laser treatment). (Note: This figure does not include all imaging time-points for this eye.) (D-F) After marking ALCS pores, mean ALCS pore geometry was quantified in central and peripheral regions (green boundaries) and in 60° sectors (fuchsia meridians). Significant increases in ALCS pore geometry were first measured in the central region and superotemporal and temporal sectors at 238 days after the initial laser treatment. (G-I) SDOCT maximum intensity projection images of the ONH showing marked ALCS points (yellow dots) from all B-scans and the BMO reference plane (red line) for the corresponding time-points in (A-C). (I) A significant increase in mean ALCSD was measured simultaneously with (F) the first significant change in pore geometry. A white asterisk (*) indicates the time corresponding to the first significantly measured change in ALCS pore or ONH geometry. Scale bar: 350 μm.

The time-points corresponding to the first significant changes in ALCS pore geometry, ONH parameters, and RNFLT were compared in each EG eye. In two EG eyes (OHT-64 and OHT-65), the first structural parameters that significantly changed from baseline were ALCS pore geometry, mean ALCSD, and mean MRW. Images of the ALCS microarchitecture and ONH acquired at baseline time-points and the time-point of first significant change in ALCS pore geometry and mean ALCSD for the EG eye of OHT-64 are shown in Fig 5. Mean ALCS pore geometry and mean ALCSD were not significantly different between baseline (Fig 5A,5D and 5G) and a subsequent time-point of no change (Fig 5B,5E and 5H) (*P*>.05). The first statistically significant change in ALCS pore geometry and mean ALCSD occurred simultaneously in this EG eye (Fig 5C,5F and 5I). Mean pore area was significantly larger relative to baseline time-points in the central region (1,174.7 ± 783.2 μm^2^ vs. 716.7 ± 440.1 μm^2^) and in the temporal sector (1,276.2 ± 859.4 μm^2^ vs. 812.4 ± 635.6 μm^2^). Corresponding local increases in mean pore NND were measured relative to baseline time-points in the central region (52.0 ± 14.8 μm vs. 39.3 ± 10.3 μm) and the superotemporal and temporal sectors (50.5 ± 16.8 μm vs. 43.0 ± 11.1 μm and 54.8 ± 10.2 μm vs. 38.4 ± 11.7 μm, respectively), while mean pore elongation significantly increased relative to baseline in the superotemporal sector (1.79 ± 0.59 vs. 1.55 ± 0.34). Similarly, the first statistically significant increase in mean ALCSD relative to baseline (354.3 μm vs. 199.5 μm, respectively) (Fig 5I) occurred simultaneously with the first significant change in ALCS pore geometry (Fig 5C and 5F).

**Fig 6:**
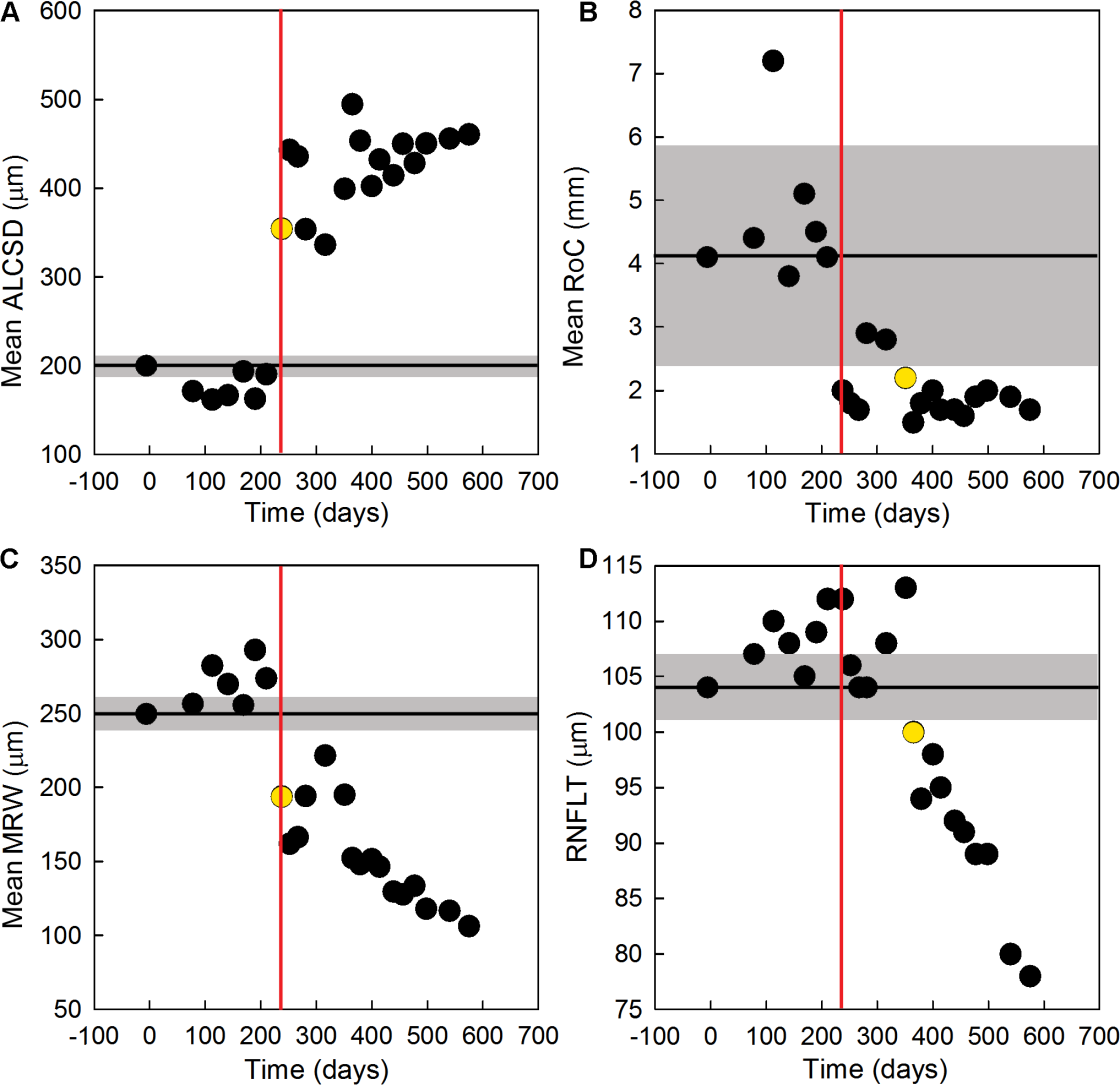
Early changes in ONH parameters, RNFLT, and ALCS pore geometry in the EG eye of monkey OHT-64. Values for (A) mean ALCSD, (B) mean RoC, (C) mean MRW and (D) RNFLT are shown as a function of study time for all measured time-points (black circles) before and after the initial laser treatment (day 0). The black horizontal line in each plot indicates the baseline value for each parameter while the gray shaded region represents the 95% confidence interval for each parameter calculated from data measured in the fellow control eye. Yellow circles represent the time-point of first significant change in each plotted parameter, while vertical red lines represent the time-point of first significant change in ALCS pore geometry. The first parameters to significantly change from baseline values were (A) mean ALCSD, (C) mean MRW, and ALCS pore geometry (238 days after the initial laser treatment), followed by a significant change in (B) mean RoC (351 days after the initial laser treatment). (D) RNFLT was the last parameter to show a significant change (365 days after the initial laser treatment).

Longitudinal changes in ONH parameters and RNFLT are shown in Fig 6 for all measured time-points in the same EG eye of monkey OHT-64. The first time-point of significant change from baseline in ALCS pore geometry (vertical red line) occurred simultaneously with the first significant change in mean ALCSD and mean MRW (yellow dots; Fig 6A and 6C). The next parameter to significantly change from baseline was mean RoC (Fig 6B). RNFLT (Fig 6D) was the last measured parameter to show an initial significant change.

**Fig 7:**
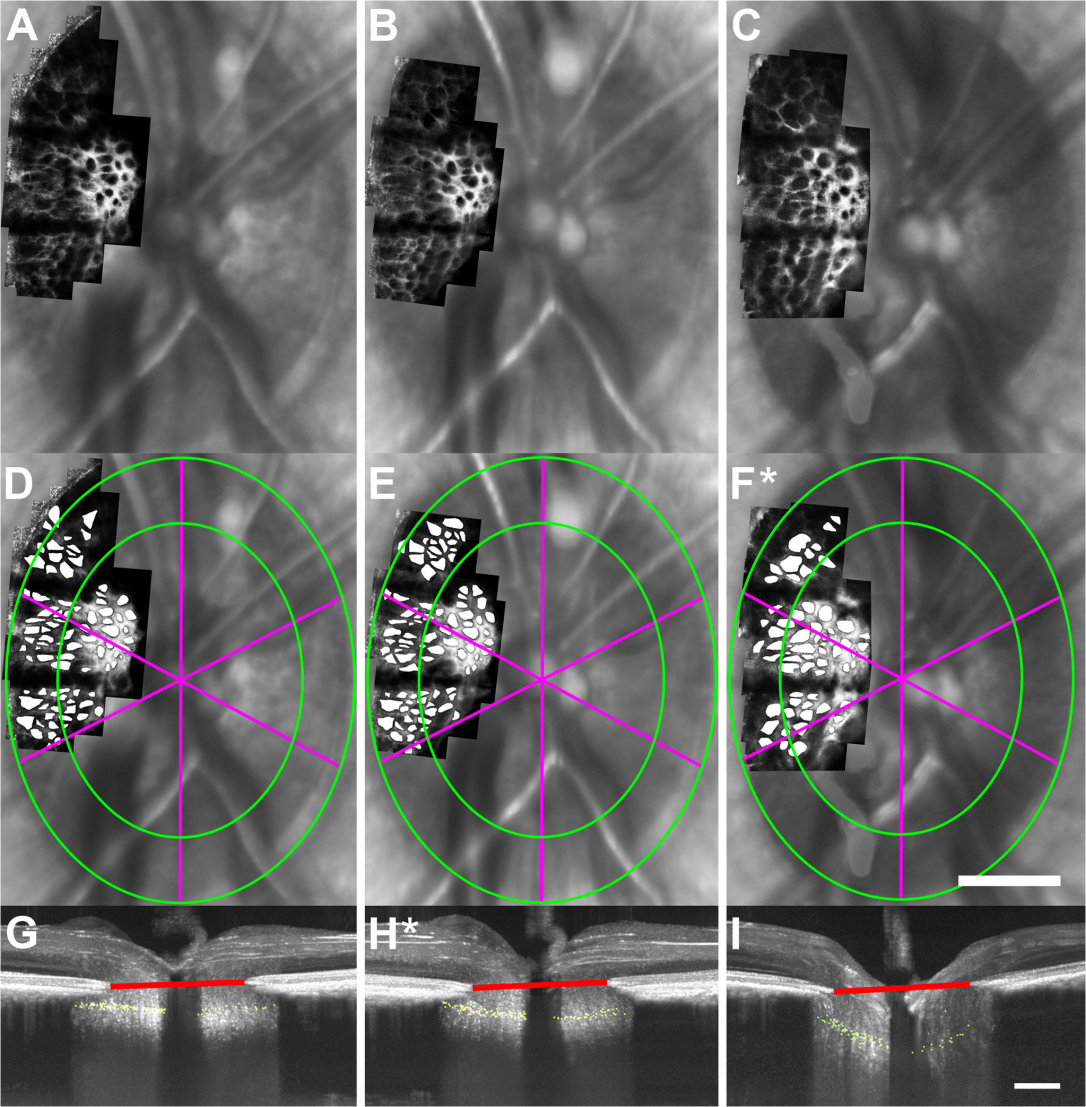
The first significant change in mean ALCSD (relative to baseline time-points) occurred prior to the first significant change in ALCS pore geometry in the EG eye of monkey OHT-66. Images of (A-C) ALCS microarchitecture and (G-I) the ONH were each acquired at baseline (left column), a later time-point (middle column, 49 days after the initial laser treatment) that showed the first statistically significant change (*) in mean ALCSD, and a subsequent time-point corresponding to the first significant change (*) in ALCS pore geometry (right-most column, 168 days after the initial laser treatment). (Note: This figure does not include all imaging time-points for this eye.) (D-F) After marking ALCS pores, mean ALCS pore geometry was quantified in central and peripheral regions (green boundaries) and in 60° sectors (fuchsia meridians). Significant changes in ALCS pore geometry were first seen in central and peripheral regions and all sectors at 168 days after the initial laser treatment. (G-I) SDOCT maximum intensity projection images of the ONH showing marked ALCS points (yellow dots) from all B-scans and the BMO reference plane (red line) for the corresponding time-points in (A-C). (H) A significant increase in mean ALCSD was measured prior to (F) the first significant change in ALCS pore geometry. A white asterisk (*) indicates the first significantly measured change in ALCS pore geometry and mean ALCSD. Scale bar: 350 μm.

In contrast to the EG eyes of monkeys OHT-64 and OHT-65 in which three structural parameters simultaneously had an initial, significant change, three EG eyes (OHT-63, OHT-66, and OHT-67) had an initial, significant change in only a single parameter. For example, the first parameter to significantly change from baseline in two EG eyes (OHT-66 and OHT-67) was mean ALCSD. Fig 7 shows images of the ALCS microarchitecture and ONH acquired at baseline (left column), the time-point of first significant change in mean ALCSD (middle column), and the time-point of first significant change in ALCS pore geometry (right column) for the EG eye of monkey OHT-66. At 49 days following the initial laser treatment in this EG eye (Fig 7, middle column), a statistically significant increase in mean ALCSD (Fig 7H) was measured relative to baseline (Fig 7G) (174.3 μm vs. 156.3 μm), while no statistically significant differences were measured in any ALCS pore parameter (Fig 7E) relative to baseline (Fig 7D) (*P>*.05). At 168 days following the initial laser treatment (Fig 7, right column), mean ALCSD further increased from 174.3 μm to 285.1 μm (Fig 7I) and the first significant change in ALCS pore geometry (Fig 7F) was measured. Mean ALCS pore area significantly increased relative to baseline time-points in the central (917.3 ± 545.4 μm^2^ vs. 703.0 ± 382.5 μm^2^, respectively) and peripheral (1,456.1 ± 864.1 μm^2^ vs. 865.7 ± 561.0 μm^2^, respectively) regions and in the superotemporal (1,548.8 ± 1029.2 μm^2^ vs. 890.7 ± 470.9 μm^2^, respectively) and temporal (1,020.0 ± 591.3 μm^2^ vs. 712.4 ± 482.5 μm^2^, respectively) sectors. Corresponding local increases in mean pore NND were measured relative to baseline in central (37.1 ± 9.8 μm vs. 33.7 ± 9.6 μm, respectively) and peripheral (46.0 ± 11.4 μm vs. 36.3 ± 8.8 μm, respectively) regions and in the superotemporal (44.9 ± 9.7 μm vs. 35.7 ± 9.3 μm, respectively) and temporal (38.6 ± 11.9 μm vs. 34.0 ± 9.6 μm, respectively) sectors, while mean pore elongation significantly decreased relative to baseline values in the temporal (1.80 ± 0.49 vs. 1.86 ± 0.79, respectively) and inferotemporal (1.36 ± 0.17 vs. 1.60 ± 0.45, respectively) sectors.

**Fig 8:**
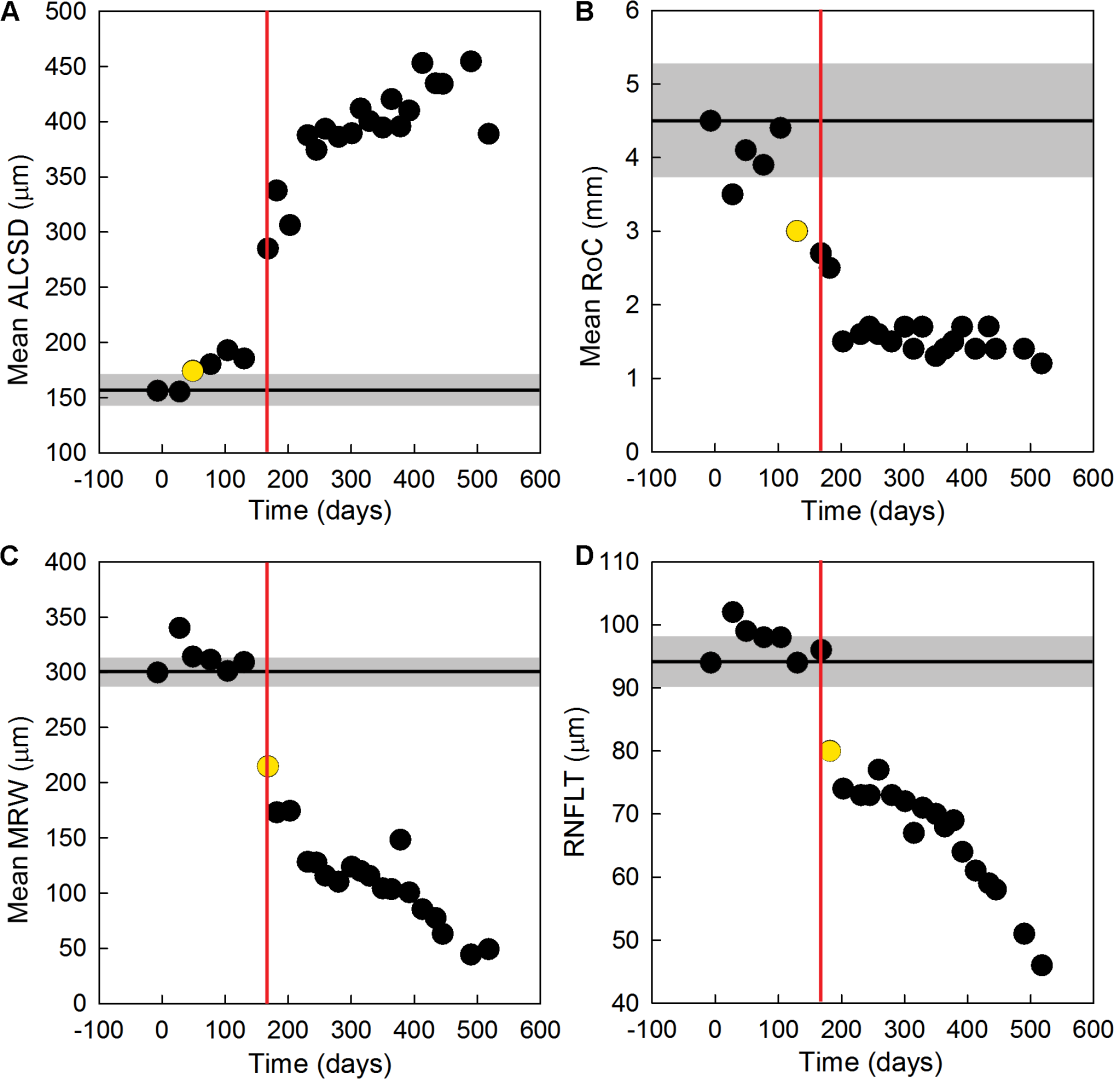
Early changes in ONH parameters, RNFLT, and ALCS pore geometry in the EG eye of monkey OHT-66. Values for (A) mean ALCSD, (B) mean RoC, (C) mean MRW, and (D) RNFLT are shown as a function of study time for all measured time-points (black circles) before and after the initial laser treatment (day 0). The black horizontal line in each plot indicates the baseline value for each parameter while the gray shaded region represents the 95% confidence interval for each parameter calculated from data measured in the fellow control eye. Yellow circles represent the time-point of first significant change in each plotted parameter, while vertical red lines represent the time-point of first significant change in ALCS pore geometry. The first parameter to significantly change from baseline values was (A) mean ALCSD (49 days after the initial laser treatment). The next parameter to significantly change from baseline was (B) mean RoC (130 days after the initial laser treatment), followed by simultaneous significant changes in ALCS pore geometry and mean MRW (168 days after the initial laser treatment). RNFLT was the last measured parameter to significantly change (182 days after the initial laser treatment).

Longitudinal changes in ONH parameters and RNFLT measured in the same EG eye of monkey OHT-66 are shown in Fig 8 for all time-points. Mean ALCSD was the first parameter to significantly change relative to baseline (Fig 8A), followed by a significant change in mean RoC (Fig 8B). ALCS pore geometry and mean MRW next exhibited their first significant changes from baseline at the same time-point (Fig 8C). Similar to results from the EG eye of OHT-64, RNFLT was the last parameter to show a first significant change from baseline (Fig 8D).

### Time-course of structural change onset

**Fig 9:**
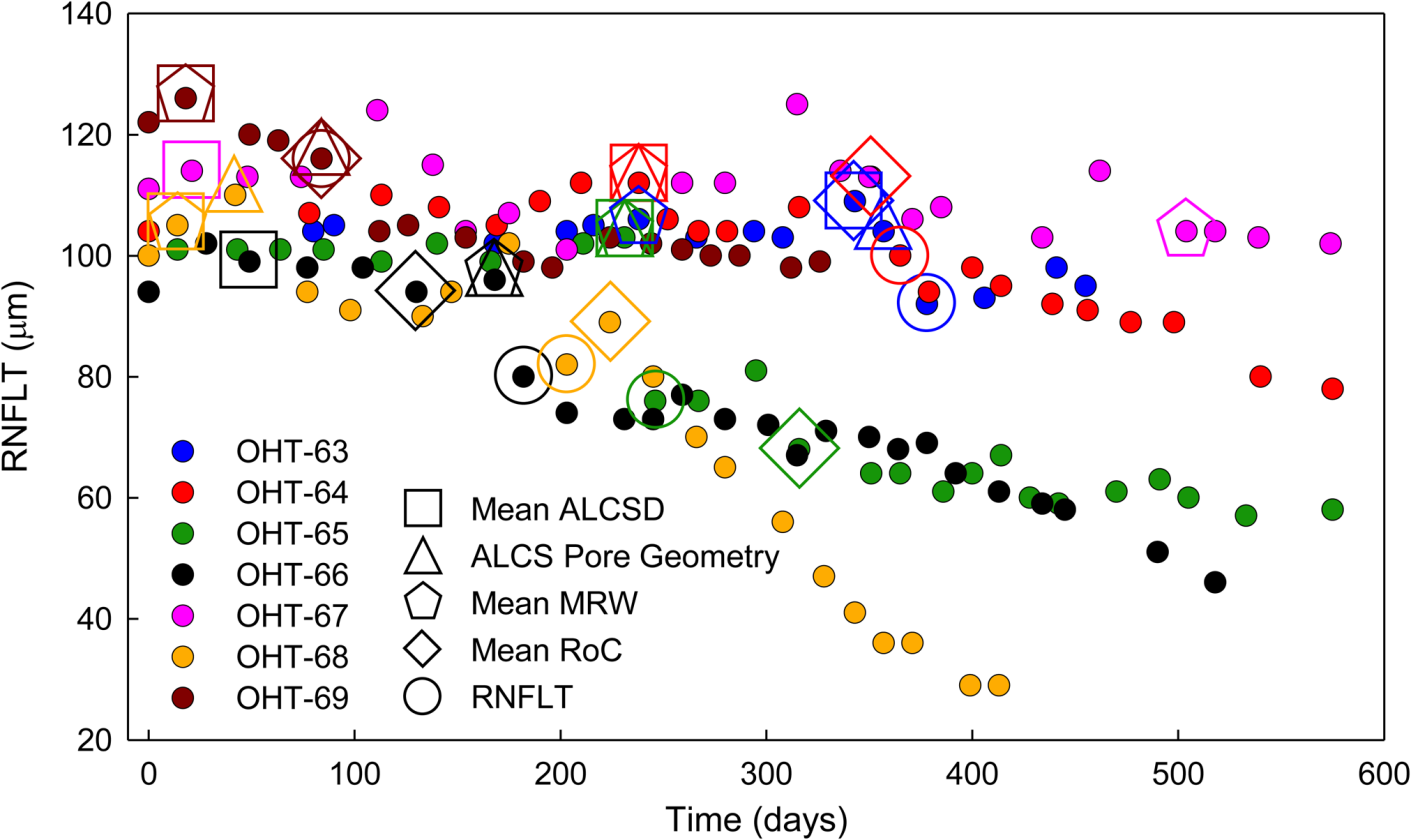
Summary of the time-course when the first significant changes were measured (relative to baseline values) in ONH parameters, ALCS pore geometry, and RNFLT in all EG eyes. RNFLT values are plotted as a function of study time for all measured time-points (filled circles, with each color representing a different EG eye). A progressive decrease in RNFLT was measured throughout the course of the study in all EG eyes except OHT-67 (which also showed no significant change in ALCS pore geometry, mean RoC, and RNFLT). Symbols representing the time-points of first significant change in mean ALCSD (square), ALCS pore geometry (triangle), mean MRW (pentagon), mean RoC (diamond) and RNFLT (circle) are overlaid on the plot and are color-coded for each EG eye. One of the first significant structural changes to be measured in EG eyes was an increase in mean ALCSD (square, 6 of 7 EG eyes). A significant decrease in mean MRW (pentagon) simultaneously accompanied this early change in 4 eyes and was the first, sole significant structural change in 1 of 7 EG eyes (OHT-63). A significant change in mean ALCS pore geometry (triangle) occurred first in 2 of 7 EG eyes (OHT-64, OHT-65).

The time-course of the first significant changes measured in ALCS pore geometry, ONH parameters, and RNFLT relative to baseline is summarized in Fig 9 and Table 7 for all 7 EG eyes. A sole change in MRW occurred first in one EG eye (OHT-63) while a sole change in mean ALCSD occurred first in two EG eyes (OHT-66 and OHT-67). Simultaneously changes in mean ALCSD and mean MRW occurred first in two EG eyes (OHT-68 and OHT-69) while simultaneous changes in mean ALCSD, mean MRW, and ALCS pore geometry occurred first in the remaining two EG eyes (OHT-64 and OHT-65). Based on these results, mean ALCSD increased first in 6 out of 7 EG eyes, with 4 eyes showing a simultaneous decrease in MRW and 2 eyes showing a simultaneous change in ALCS pore geometry. For six EG eyes in which statistically significant changes were measured in all structural parameters by the end of the study, RNFLT was the last parameter to change in 3 EG eyes while mean RoC was the last parameter to significantly change in 2 EG eyes. Simultaneously significant changes in mean RNFLT and mean RoC were the last changes to occur in 1 EG eye. Kaplan-Meier curves and log-rank analyses determined that the relative timing of the first change in mean ALCSD was statistically significantly different (i.e., occurred earlier) from the first measured change in RNFLT and the first change in mean RoC across all eyes (*P* = .05). No other parameters exhibited statistically significant differences in the temporal sequence of onset of change from each other across eyes.

**Table 7:** Days post-laser of first significant change in mean ALCSD, mean MRW, ALCS pore geometry, mean RoC, and RNFLT ordered by mean onset position.

Animal ID	Mean ALCSD	Mean MRW	ALCS Pore Geometry	Mean RoC	Mean RNFLT
OHT-63	343 (2)	238 (1)	357 (3)	343 (2)	378 (4)
OHT-64	238 (1)	238 (1)	238 (1)	351 (2)	365 (3)
OHT-65	231 (1)	231 (1)	231 (1)	316 (3)	246 (2)
OHT-66	49 (1)	168 (3)	168 (3)	130 (2)	182 (4)
OHT-67	21 (1)	504 (2)	—	—	637 (3)
OHT-68	14 (1)	14 (1)	42 (2)	224 (4)	203 (3)
OHT-69	18 (1)	18 (1)	84 (2)	84 (2)	84 (2)
Mean Onset Position	1.1 (1)	1.4 (2)	2.0 (3)	2.5 (4)	3.0 (5)

‘()’—Position of change relative to all other parameters’ positions of change in the analyzed eye.

‘—’—No statistically significantly change was measured at any point in the study (*P*>.05).

## Discussion

The purposes of this study were to (1) examine inter-eye differences in ONH structure and anterior lamina cribrosa surface (ALCS) pore geometry *in vivo* in fellow eyes of bilaterally normal monkeys and (2) characterize the time-course of *in vivo* changes in ALCS pore microarchitecture concurrent with structural changes in the ONH and RNFL in early EG. *In vivo* measures of ALCS pore geometry and ONH structure were similar between fellow eyes of normal non-human primates. The results of a cluster analysis performed in normal eyes often correlated with the results of local pore comparisons/analyses performed in each eye. For example, a larger degree of spatial clustering of pores according to their area was noted in the right eye of monkey M067 (panel C, S2 Fig) in which larger area (red) pores were more clumped in the superotemporal portion of the LC. This increased amount of spatial clustering not only agreed with the high positive Moran’s I value of +0.11, but also spatially agreed with the fact that the superotemporal sector contained pores with statistically significantly larger areas and NNDs (compared to the temporal and inferotemporal sectors) in the same eye. Likewise, the fact that pore area was statistically similar across all sectors in the right eyes of monkeys M071 and M070 also agreed with the more random distribution of pore areas in the same eyes (as reflected by the low Moran’s I values of -0.02 and -0.01, respectively; panels A,B in S2 Fig).

In the monkey model utilized in this study, IOP was elevated unilaterally (with the fellow eye serving as a control) via a gradual laser ablation of the trabecular meshwork. The maximum IOP when averaged across all 7 EG eyes was 50.4 ± 4.8 mmHg (Table 3), representing a +282% mean difference in the maximum IOP experienced by our EG eyes relative to mean IOP values in control eyes (13.2 ± 1.4 mmHg). This average value of maximum IOP measured in our EG eyes is comparable to those reported in other studies. For example, Fortune et al. reported an average peak IOP of 41.0 ± 10.7 mmHg in EG monkey eyes compared to a mean IOP of 10.8 ± 1.7 mmHg in control eyes, reflecting a +280% mean difference in maximum IOP values relative to mean values in controls [48]. Similarly, Downs et al. measured a +241% mean difference between maximum IOP values in EG monkey eyes and mean IOP values in fellow control eyes [49]. Due to the fact that IOP levels can become high in the monkey model of experimental glaucoma, it is important to lower IOPs in EG eyes to “normal” levels when imaging the ONH to examine chronic changes in ONH and LC structure that result from sustained elevations in IOP. Prior to *in vivo* imaging, IOPs were pharmacologically reduced in our EG eyes to levels that were, on average, only 6 mmHg higher than mean IOP values in fellow control eyes (Table 3). Imaging ONH and LC parameters in EG eyes with pressures that were reduced nearly to normal values better ensured that the structural changes measured in the ONH and LC were the result of a chronic elevation in IOP and not a temporary or acute spike in pressure.

*In vivo* measurements of mean ALCSD and LC microarchitecture made in our normal control eyes and EG eyes at baseline are similar to values reported in previous studies. The average value of mean ALCSD calculated across all control eyes was 214.0 ± 25.3 μm and is comparable to earlier *in vivo* measurements of mean ALCSD made in normal monkey eyes (~ 220–230 μm) [21,50,51]. Average values of mean ALCS pore geometry measured at baseline in this study across all control eyes (mean pore area = 871.9 ± 181.8 μm^2^; mean pore elongation = 1.69 ± 0.05; mean pore NND = 37.7 ± 6.5 μm) and across all EG eyes (mean pore area = 838.0 ± 130.1 μm^2^; mean pore elongation = 1.71 ± 0.13; mean pore NND = 37.2 ± 2.6 μm) are similar to previous *in vivo* measurements of LC pore geometry in normal non-human primate eyes [6,34].

While the coefficients of variation in RNFLT, mean ALCSD, and mean MRW were small in all control eyes throughout the experiment (<6.5%), the average coefficient of variation in mean RoC across all control eyes was larger (21.7 ± 7.6%) (Table 4). One potential reason for the increased variability in mean RoC in control eyes is that the ALCS tends to be relatively flat in normal monkey eyes [37] and RoC measurements are likely prone to higher variability when assessed on flatter surfaces. For example, values of mean RoC can be rather large for flat surfaces (approaching infinity for a perfectly flat surface). Therefore, small variations in the degree of LC “flatness” could have a large impact in the mean value of RoC measured at a given time-point and potentially result in greater variability when measured over time in normal eyes. Additionally, the amount of prelaminar tissue in the ONH is typically thickest at the edge of the neuroretinal rim, making it more challenging to visualize and quantify the surface curvature of the ALCS toward the periphery of the ONH in normal eyes and potentially increasing measurement variability when examining the same normal eyes at multiple time-points. Despite the relatively increased variability in mean RoC in control eyes, we believe it is still important to examine changes in mean RoC in combination with changes in mean ALCSD to better characterize the shape of the ALCS, especially in EG eyes. The use of a single parameter (such as mean ALCSD) provides only a partial characterization of the ALCS as it is possible to have multiple anterior LC surfaces with very different mean RoCs (indicating differently curved surfaces) but the same mean ALCSD [37].

Significant changes in ONH parameters and RNFLT were measured throughout the study in EG eyes. At time-points corresponding to the first significantly measured change in each parameter relative to baseline values, there was a +38% average increase in mean ALCSD (i.e., a more posteriorly displaced ALCS), -55% average decrease in mean RoC (i.e., a more steeply curved ALCS), and -19% and -13% average decreases in mean MRW and RNFLT (i.e., a loss of RNFL axons), respectively, across all EG eyes. These initial changes further increased during the progression of experimental glaucoma. At the final imaging time-points for each monkey, we measured a 133% average increase in mean ALCSD (i.e., an even more posteriorly-displaced ALCS), -70% average decrease in mean RoC (i.e., an even more steeply curved ALCS), and -61% and -34% average reductions in mean MRW and RNFLT (i.e., an increased loss of RNFL axons), respectively, across all EG eyes.

In addition to measuring longitudinal changes in ONH and RNFL structure, we report the first *in vivo* longitudinal changes in LC microarchitecture in non-human primates with experimental glaucoma. Changes in ALCS pore parameters were measured locally, but not globally during EG. A fair comparison of global ALCS pore changes over time would require analysis of an ALCS area that was visible at all imaging time-points. The size of such an area would depend on the amount of lamina imaged at each time-point and could potentially be too small for a meaningful global analysis if the area imaged at any one time-point was smaller than that imaged at other time-points (e.g., the smaller area in Fig 5C vs. that in Fig 5A). Therefore, we analyzed local changes in ALCS pore parameters only for sectors or regions that were present at the given follow-up time-point and at baseline. Significant increases were measured in mean ALCS pore area at the first time-point of change (relative to baseline) in the temporal sector (+35.5%) and in central (+23.6%) and peripheral (+29.9%) regions (Table 6). Corresponding increases in mean ALCS pore NND were also measured at the first time-point of significant change in the superotemporal (+13.2%) and temporal (+17.1%) sectors, as well as in central (+11.6%) and peripheral (+22.4%) regions, while no statistically significant changes were measured in pore elongation for any regions or sectors. It is important to note that measuring a change in pore NND in isolation (i.e., without measuring concurrent changes in pore area or elongation) might be of limited value for describing beam and pore remodeling. For example, a change in the center-to-center separation between adjacent pores (pore NND) could result from a change in pore area (with no change in beam diameter), a change in beam diameter (with no change in pore area), or a change in pore area and beam diameter. However, knowledge of the spatial arrangement of pores and beams can be improved when combining changes in pore NND with changes in other pore parameters. In this study, the increases in pore NND and area measured at the time-point of first significant change in ALCS pore geometry indicate that ALCS pores became larger and further separated temporally, centrally and peripherally in early EG. The degree to which a change in laminar beam diameter also contributes to the increase in pore NND will be subject to future study.

The first statistically significant changes in ALCS pore geometry measured in our study (i.e., increased pore area and NND) are similar to differences in ALCS pore geometry described in glaucomatous eyes in recent cross-sectional studies. Vilupuru et al. acquired *in vivo* images of ALCS pores at a single time-point in 3 monkey eyes with moderate to advanced stages of experimental glaucoma (i.e., mean deviations > -6.5 dB) and found statistically significantly increased values of mean ALCS pore area in all EG eyes, pore elongation in 1 EG eye, and NND in 2 EG eyes (relative to fellow control eye values) [6]. Similar to our finding of no significant change in pore elongation at the first time-point of significant change in ALCS pore geometry, Wang et al. reported no significant differences in LC pore aspect ratio (or elongation) between healthy and glaucomatous human patients measured at a single time-point [38].

When characterizing the time-course of first significant change in RNFLT, ONH parameters and ALCS microarchitecture, we found that mean ALCSD, mean MRW, and mean ALCS pore geometry (quantified globally and locally) were always among the first 3 parameters to change in our EG eyes. Mean ALCSD was the first parameter (or one of the first parameters) to statistically significantly change from baseline in 6 of 7 EG eyes. Statistical analyses showed that the relative timing of the first measured change in mean ALCSD was significantly different from the first measured change in RNFLT and in mean RoC, suggesting mean ALCSD occurred at a substantially earlier time-point. Furthermore, mean MRW simultaneously decreased in 4 of these 6 eyes and was the sole parameter to first change in the 7^th^ EG eye. Conversely, RNFLT was the last or second to last parameter to change in 6 EG eyes. (No significant change in RNFLT was measured in the 7^th^ EG eye, OHT-67, throughout the entire study duration.) These results agree with previous studies that found mean ALCSD and mean MRW to change prior to a change in RNFLT. Strouthidis et al. measured an increase in mean ALCSD prior to a reduction in RNFLT in 9 monkey eyes with unilateral experimental glaucoma [50]. In addition, He et al. found that changes in mean ALCSD and MRW occurred prior to the first detectable loss in RNFLT or loss in multifocal electroretinogram (mfERG) measures of visual function in 8 monkey eyes with unilateral experimental glaucoma [24]. In combination with our study, these works collectively suggest that mean MRW could potentially represent an improved and more sensitive surrogate marker for axonal change from glaucoma compared to RNFLT. A more focused investigation on the temporal sequence of structural changes as potential predictors of earlier disease progression is a natural extension of this work and deserving of a future study. Moreover, the results of our study build on the aforementioned studies by providing the first characterization of the onset of changes in ALCS pore microarchitecture in combination with changes in ONH parameters and RNFLT.

The first significant change in ALCS pore geometry was measured *after* the time-point of first significant change in mean ALCSD in 4 of 6 EG eyes. (No significant change in ALCS pore geometry was measured relative to baseline in one EG eye.) Several reasons could potentially account for the fact that changes in mean ALCSD and mean ALCS pore parameters did not occur simultaneously in more EG eyes. First, a uniform posterior displacement of the LC surface (i.e., an increase in mean ALCSD with minimal change in mean RoC) could result in an ALCS shape that is relatively unaltered from baseline conditions with a negligible change in ALCS pore geometry. Second, overlying features (such as vasculature and the neuroretinal rim) obstruct our ability to view the most superior, inferior, and peripheral ALCS beams and pores. Given our inconsistent ability to image the nasal side of the ONH at all longitudinal time points and the fact that several studies have found early losses in RNFLT to occur in superior- and inferior-temporal regions of the ONH in glaucoma, we focused our analyses on ALCS microarchitecture in the temporal half of the disk. Approximately 25% of the ALCS within the BMO ellipse was imaged, on average, in normal and EG eyes in this study. (When imaging the temporal side of the disk, one might expect to maximally image ~45% of the ALCS given that the major vessels from the central retinal artery and vein usually run in a vertical direction along the middle of the ONH in monkey eyes.) Consequently, LC pores were typically visualized over more central/mid-peripheral regions of the ALCS, resulting in an increased number of pores that were imaged along the flatter, more central portion of the ALCS compared to the more steeply sloped portions of the ALCS at the periphery of the LC. Therefore, we likely did not observe changes in the most peripherally located ALCS pores within the ONH where one might expect to see even larger changes in pore geometry associated with a posterior movement in the ALCS in early EG. Third, it is possible that LC pore geometry did not significantly change at the ALCS, but was potentially altered at a greater depth than was visualized in this study. In future studies, it will be important to image and examine LC pore microarchitecture at various LC depths at baseline and follow-up time-points in order to fully characterize changes in beams and pores throughout the entire depth of the LC in early experimental glaucoma.

Finally, some EG eyes demonstrated more rapid changes in ONH and LC parameters compared to other EG eyes. For example, early changes in ONH and LC structure occurred very quickly in the EG eye of monkey OHT-69, as the first significant changes in ONH parameters (mean ALCSD, RoC, and MRW), mean ALCS pore parameters, and RNFLT occurred within 84 days of the initial laser treatment (Fig 9). Conversely, early changes in ONH and LC structure occurred very slowly (or not at all) in the EG eye of monkey OHT-67. While an initial significant change in mean ALCSD was measured 21 days after the initial laser treatment, this EG eye showed an initial significant change in mean MRW 504 days after the initial laser treatment with no statistically significant changes in RNFLT, mean RoC, and ALCS pore microarchitecture for the entire study (Fig 9). A possible reason for this difference in progression rates could be due to different compliance levels of the LC between eyes. The fact that the EG eye of monkey OHT-67 had a significant initial increase in mean ALCSD but no significant change in mean RoC or ALCS pore geometry suggests that the LC migrated posteriorly in the neural canal without significantly changing its surface shape (i.e., a more uniform posterior displacement), potentially as a result of an increased compliance (or decreased resistance) of the LC after chronic exposure to elevated IOP. The resistance to change demonstrated by the LC exhibited in the EG eye of monkey OHT-67 was not observed in the EG eye of monkey OHT-69, in which significant initial increases were measured soon after the initial laser treatment in all LC-related parameters (mean ALCSD, mean RoC, and mean ALCS pore parameters).

In conclusion, while ONH and ALCS pore parameters were similar between fellow eyes of normal monkeys, dramatic changes in mean ALCSD, mean MRW and ALCS beams and pores occurred in early stages of EG. Moreover, a statistically significant structural change in mean ALCSD was found to precede a significant loss in RNFLT across EG eyes. The ability to jointly examine structural changes in the ONH and LC microarchitecture can be used to better understand biomechanical remodeling of the neural canal and LC on global and local spatial scales in response to chronically elevated intraocular pressure.

## Supporting Information

S1 Fig SDOCT maximum intensity projection images from all radial B-scans acquired in right and left eyes of 6 normal monkeys. Marked ALCS points from all scans are shown using orange dots and the BMO reference plane is represented using a red line. Mean ALCSD and mean RoC did not differ significantly between fellow eyes of all 6 monkeys (*P*>.05). Scale bar: 300 μm.
(TIF)

S2 Fig Images illustrating the variability in the spatial arrangements of marked ALCS pores (as classified by their area) in 3 monkey eyes. Green lines represent the BMO ellipse. Pores were classified as having large (red), medium (blue), and small (yellow) areas in each eye via cluster analysis. (A) Pores in the right eye of monkey M071 had the largest negative spatial autocorrelation of all eyes based on pore area, though it was not statistically significant (Moran’s I index = -0.02, *P* = .97). (B) Pores in the right eye of monkey M070 had a non-statistically significant negative spatial autocorrelation based on their area (Moran’s I index = -0.01, *P* = .54), indicating a slightly more random spatial distribution of pores according to their area. (C) Pores in the right eye of monkey M067 had the largest positive spatial autocorrelation of all eyes based on pore area that was also statistically significant (Moran’s I index = 0.11, *P* < .05). A higher degree of spatial clustering of pores with similar area (or color) was observed in this eye. Across all eyes, significant positive spatial autocorrelations in pore area (*P* < .05) were found in 6 of 12 eyes, where pores of similar area tended to be more spatially clustered. The remaining 6 eyes had non-significant Moran’s I indices that were approximately 0, indicating that pores were more randomly arranged based on their area.
(TIF)

S3 Fig ALCS microarchitecture from the temporal side of a representative control eye at different time-points. (A,B) AOSLO montages of the ALCS overlaid on the corresponding SLO images in the control eye of monkey OHT-64 at two time-points separated by 119 days. ALCS structure appears subjectively similar between imaging sessions. (C,D) Pores were manually marked in each AOSLO montage (white filled shapes). 114 pores were analyzed at both time-points. No statistically significant differences in ALCS pore geometry were measured between time-points on global and local levels (*P*>.05), except for pore area in the inferotemporal sector (*P <* .05). These results agree with earlier findings from our previous study that revealed small intersession variability in pore geometry in normal rhesus monkeys [34]. (E,F) SDOCT maximum intensity projection images from all B-scans acquired at the same time-points as the AOSLO images in (A,B). The ALCS (yellow dots) and BMO reference plane (red lines) were manually marked in each SDOCT B-scan. No statistically significant difference in mean ALCSD was measured between the two time-points [215.2 μm (E) vs. 220.6 μm (F)]. Scale bar: 350 μm.
(TIF)

S1 Table Moran’s I index values obtained from spatial autocorrelation analyses in both eyes of 6 bilaterally normal monkeys. (DOCX)
