# Supplementary figures and images for: In Vivo Changes in Lamina Cribrosa Microarchitecture and Optic Nerve Head Structure in Early Experimental Glaucoma
Source: PLoS One. 2015 Jul 31;10(7):e0134223. doi: 10.1371/journal.pone.0134223 (PMC4521723; doi:10.1371/journal.pone.0134223)

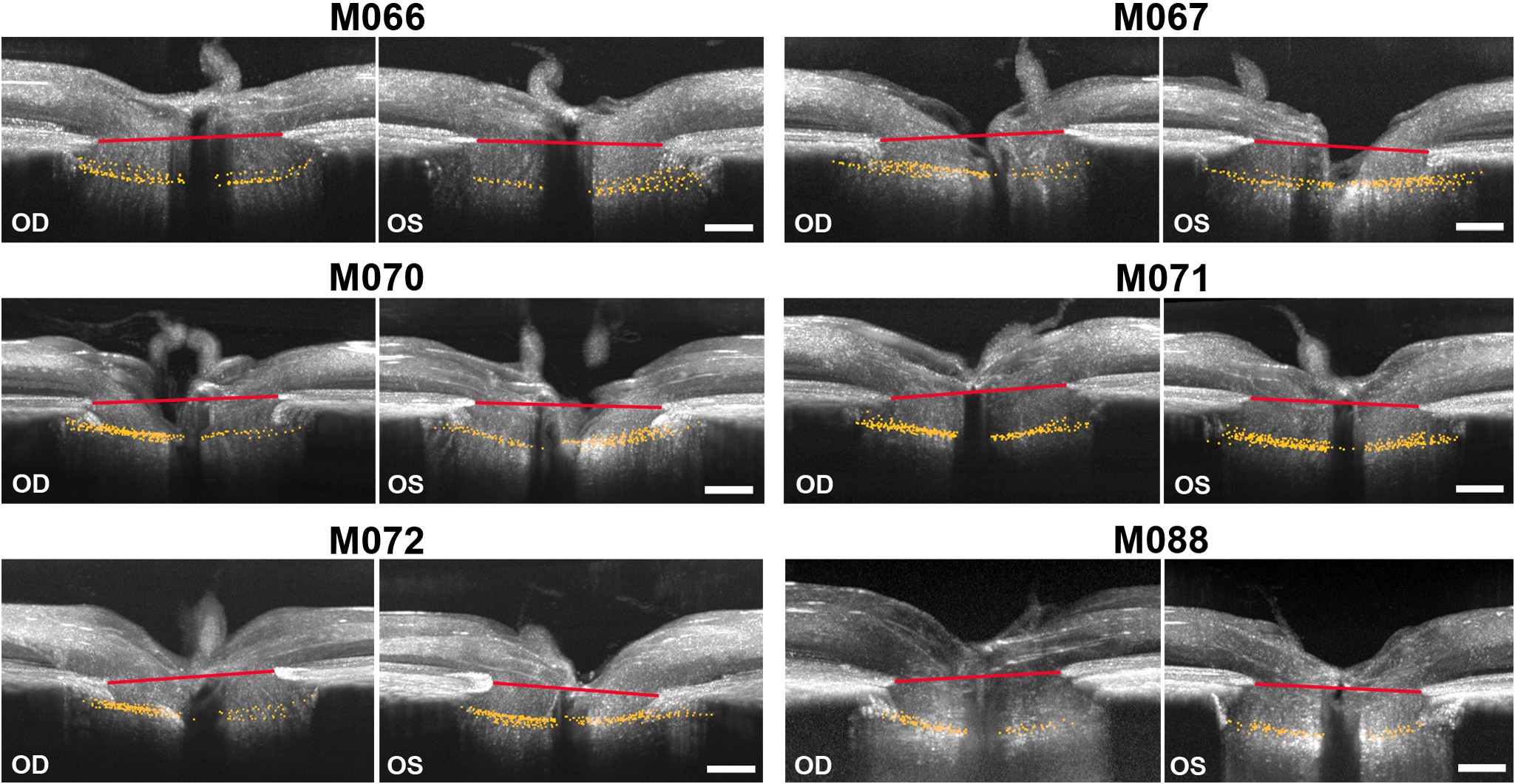

Supplement: S1 Fig — Marked ALCS points from all scans are shown using orange dots and the BMO reference plane is represented using a red line. Mean ALCSD and mean RoC did not differ significantly between fellow eyes of all 6 monkeys (P>.05). Scale bar: 300 μm. (TIF) [file pone.0134223.s001.tif]

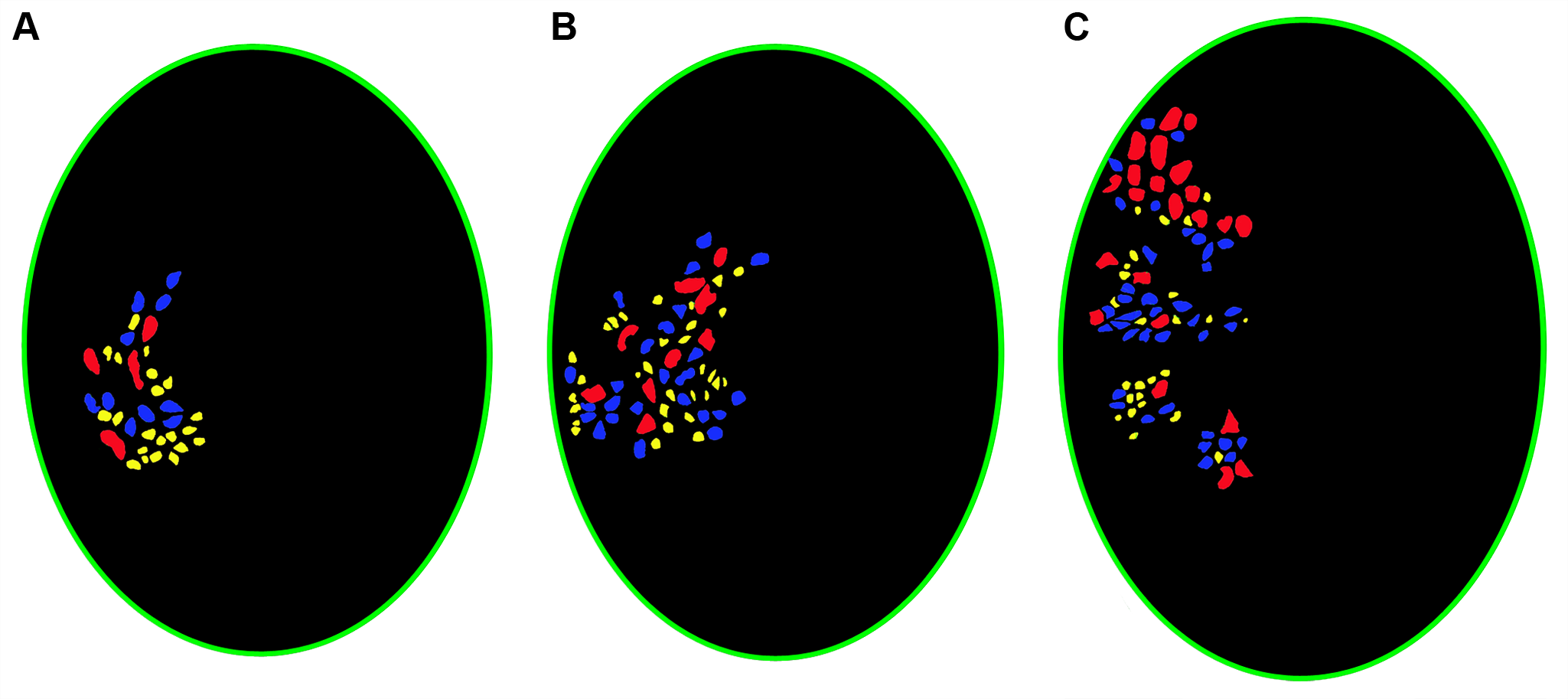

Supplement: S2 Fig — Green lines represent the BMO ellipse. Pores were classified as having large (red), medium (blue), and small (yellow) areas in each eye via cluster analysis. (A) Pores in the right eye of monkey M071 had the largest negative spatial autocorrelation of all eyes based on pore area, though it was not statistically significant (Moran’s I index = -0.02, P = .97). (B) Pores in the right eye of monkey M070 had a non-statistically significant negative spatial autocorrelation based on their area (Moran’s I index = -0.01, P = .54), indicating a slightly more random spatial distribution of pores according to their area. (C) Pores in the right eye of monkey M067 had the largest positive spatial autocorrelation of all eyes based on pore area that was also statistically significant (Moran’s I index = 0.11, P < .05). A higher degree of spatial clustering of pores with similar area (or color) was observed in this eye. Across all eyes, significant positive spatial autocorrelations in pore area (P < .05) were found in 6 of 12 eyes, where pores of similar area tended to be more spatially clustered. The remaining 6 eyes had non-significant Moran’s I indices that were approximately 0, indicating that pores were more randomly arranged based on their area. (TIF) [file pone.0134223.s002.tif]

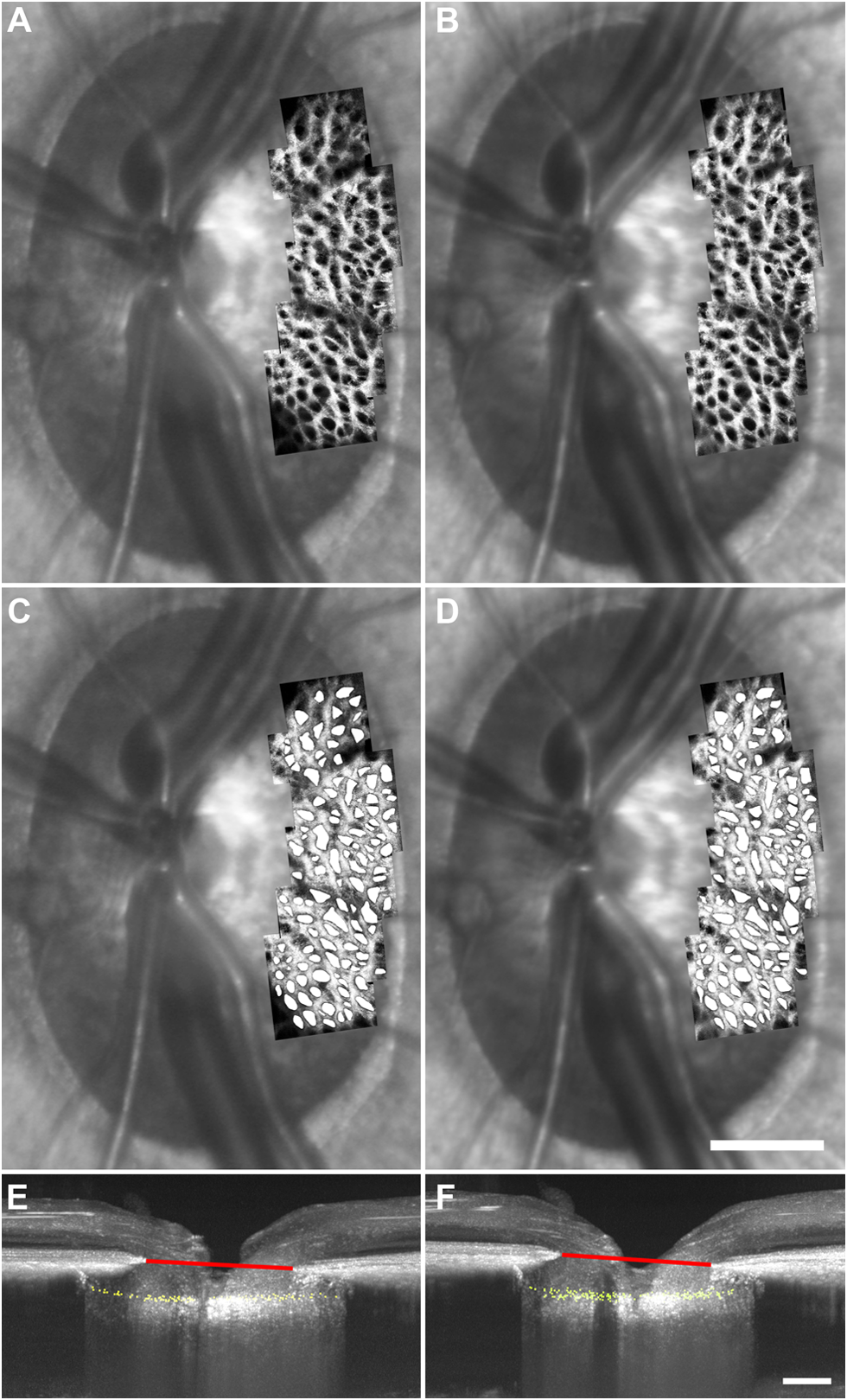

Supplement: S3 Fig — (A,B) AOSLO montages of the ALCS overlaid on the corresponding SLO images in the control eye of monkey OHT-64 at two time-points separated by 119 days. ALCS structure appears subjectively similar between imaging sessions. (C,D) Pores were manually marked in each AOSLO montage (white filled shapes). 114 pores were analyzed at both time-points. No statistically significant differences in ALCS pore geometry were measured between time-points on global and local levels (P>.05), except for pore area in the inferotemporal sector (P < .05). These results agree with earlier findings from our previous study that revealed small intersession variability in pore geometry in normal rhesus monkeys [34]. (E,F) SDOCT maximum intensity projection images from all B-scans acquired at the same time-points as the AOSLO images in (A,B). The ALCS (yellow dots) and BMO reference plane (red lines) were manually marked in each SDOCT B-scan. No statistically significant difference in mean ALCSD was measured between the two time-points [215.2 μm (E) vs. 220.6 μm (F)]. Scale bar: 350 μm. (TIF) [file pone.0134223.s003.tif]
