# Supplementary material for: In Vivo Changes in Lamina Cribrosa Microarchitecture and Optic Nerve Head Structure in Early Experimental Glaucoma
Source: PLoS One. 2015 Jul 31;10(7):e0134223. doi: 10.1371/journal.pone.0134223 (PMC4521723; doi:10.1371/journal.pone.0134223)
Supplement: S1 Table — (DOCX) [file pone.0134223.s004.docx]

**S1 Table. Moran’s I index values obtained from spatial autocorrelation analyses in both eyes of 6 bilaterally normal monkeys.**

|  | Pore Area | | Pore Elongation | | Pore NND | |
| --- | --- | --- | --- | --- | --- | --- |
| Monkey | OD | OS | OD | OS | OD | OS |
| M066 | 0.01 | 0.08* | 0.02* | 0.01 | 0.04* | 0.06* |
| M067 | 0.11* | -0.02 | 0.03* | 0.01 | 0.20* | 0.01 |
| M070 | -0.01 | 0.01 | 0.05* | 0.01 | 0.13* | 0.06* |
| M071 | -0.02 | 0.09* | 0.03* | 0.04* | 0.09* | 0.04* |
| M072 | 0.01 | 0.04* | 0.02* | -0.02 | 0.05* | 0.03* |
| M088 | 0.05* | 0.03* | 0.00 | 0.02* | 0.08* | 0.11* |

‘*’ - Statistically significant spatial autocorrelation (*P*<.05).
